# Percolation channels: a universal idea to describe the atomic structure and dynamics of glasses and melts

**DOI:** 10.1038/s41598-017-16741-3

**Published:** 2017-11-28

**Authors:** Charles Le Losq, Daniel R. Neuville, Wenlin Chen, Pierre Florian, Dominique Massiot, Zhongfu Zhou, George N. Greaves

**Affiliations:** 1 Géomatériaux, CNRS-IPGP, Paris Sorbonne Cité, 1 rue Jussieu, 75005 Paris, France; 2 Research School of Earth Sciences, The Australian National University, Mills Road, Building 142, Acton, ACT 2601 Australia; 3 Department of Mathematics and Physics, Aberystwyth University, Physical Sciences Building, Aberystwyth, Ceredigion, SY23 3BZ UK; 4 CNRS, Université d’Orléans, UPR3079 CEMHTI, 1D avenue de la Recherche Scientifique, 45071 Orléans cedex2, France; 5 University of Cambridge, Department of Materials Science & Metallurgy, Cambridge, CB3 0FS UK; 6 State Key Laboratory of Silicate Materials for Architectures, Wuhan University of Technology, Wuhan, 430070 China

## Abstract

Understanding the links between chemical composition, nano-structure and the dynamic properties of silicate melts and glasses is fundamental to both Earth and Materials Sciences. Central to this is whether the distribution of mobile metallic ions is random or not. In silicate systems, such as window glass, it is well-established that the short-range structure is not random but metal ions cluster, forming percolation channels through a partly broken network of corner-sharing SiO_4_ tetrahedra. In alumino-silicate glasses and melts, extensively used in industry and representing most of the Earth magmas, metal ions compensate the electrical charge deficit of AlO_4_^−^ tetrahedra, but until now clustering has not been confirmed. Here we report how major changes in melt viscosity, together with glass Raman and Nuclear Magnetic Resonance measurements and Molecular Dynamics simulations, demonstrate that metal ions nano-segregate into percolation channels, making this a universal phenomenon of oxide glasses and melts. Furthermore, we can explain how, in both single and mixed alkali compositions, metal ion clustering and percolation radically affect melt mobility, central to understanding industrial and geological processes.

## Introduction

Alumino-silicate melts represent the majority of the Earth’s molten rocks, and are fundamental starting materials from which technical glasses are manufactured in industry. For instance, the viscosity and density of alumino-silicate melts determine the magma buoyancy in Earth mantle and crust, and, as such, largely influence the eruption of molten rocks at Earth’s surface. In parallel, the hardness and optical refractive index of glasses formed by rapid quench of alumino-silicate melts are economically-valuable properties for technological glasses used in personal electronic devices or optical fibers, for instance.

Melt and glass properties are actually determined by how their chemical composition controls their molecular structure. Such interplay and its importance in defining key geoscience and industrial properties explains why the first model of glass structure, the Continuous Random Network (CRN) model, dates back from 1932^1^. It represents the molecular structure of simple oxide glass formers like silica, as an aperiodic disordered network of corner-sharing tetrahedra forming rings and cages. Later findings indicated that such a simple model cannot describe the structure of melts and glasses containing modifying metal cations. Indeed, as described by the Modified Random Network (MRN) model^2,3^, metal cations (Na^+^, K^+^, Ca^2+^, Mg^2+^…) break inter-tetrahedral bonds and segregate into clusters, which eventually become continuous channels once their concentration reaches the percolation threshold^2–9^. The MRN model allows silicate melts to be described in Al-free systems, like the MgO-CaO-SiO_2_ system important for the Earth mantle^10^ or the MgO-CaO-Na_2_O-SiO_2_ system important for the production of Flat window glass.

Addition of aluminum introduces another level of complexity, Al^3+^ entering mostly in tetrahedral coordination as AlO_4_^−^ in silicates, with apical oxygen atoms presenting electrical charge deficits. As a result, part of the metal cations that are present in the melt compensate this electrical charge deficit as they do in the crystalline state. In most alumino-silicates compositions, of which the substantial field of feldspars is typical, the concentration of metal cations is just sufficient to charge compensate all the AlO_4_^−^ present. The Compensated Continuous Random Network (CCRN) model^11^ accounts for these effects in the glassy state by locating compensating metallic ions in the vicinity of AlO_4_^−^. Both the MRN and CCRN models are based on the fact that metal cations occupy well-defined sites, similar to those in crystalline structures, as observed from neutron scattering and X-ray spectroscopy for example. By juxtaposing these sites with the alumino-silicate tetrahedral network, metal cations could potentially segregate into nano-structured regions^6,11^. Such regions, rich in metallic ions, would therefore influence ionic diffusion pathways and molecular configurations, and their presence may thus have important consequences for the thermo-physical properties of alumino-silicate glasses and their corresponding melts.

In particular, metal cation percolation channels in glasses may have important consequences for the Mixed-Alkali Effect (MAE), observed when different metal cations in a glass interfere non-linearly with the mobility of each other(s)^6–8,11–13^. This results, for instance, in depressing the viscosity of silicate melts and the ionic conductivity of silicate glasses^12^. Recent explanations of the MAE^8,9,11,13^ in such systems include nano-structured channels, as depicted in the MRN model, where diffusing ions of different size block one another’s passage. So far, however, this phenomenon has not yet been identified or modeled across the extensive range of alumino-silicate systems. This is the thrust of the present study, which shows that clustering and channeling of metal cations is universal in the majority of oxide melts and glasses, offering common nano-structural explanations for thermo-physical and transport properties.

## Results

### Non-linear viscosity of mixed alkali alumino-silicates and atomic structure

**Figure 1:**
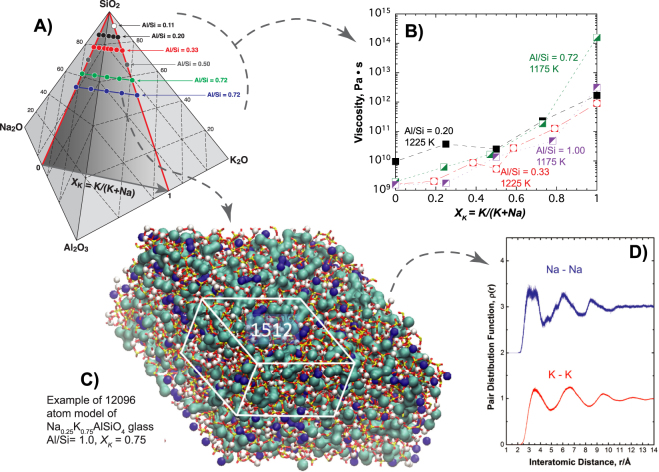
Viscosity variations measured in compensated alumino-silicate glasses and CCRN Molecular Dynamics model of Na_0.25_K_0.75_AlSiO_4_ glass demonstrating nano-segregation of alkalis. (**A**) Diagram K_2_O-Na_2_O-Al_2_O_3_-SiO_2_ in mol % showing the studied compositions. *X*_*K*_ = *K*_2_ *O/*(*K*_2_ *O* + *Na*_2_ *O*). Values and ratios are listed in Table S1. (**B**) Viscosity at constant temperature of the compensated glasses as a function of their *X*_*K*_ ratios. See Tables S1, S2, S3 and Fig. S3 for chemical compositions and viscosity data; errors are smaller than symbols. (**C**) 12,096 atom MD model of the Na_0.25_K_0.75_AlSiO_4_ glass, illustrating nano-segregation of charge compensating alkalis in percolation pathways. The white frame compares the size of the 1512 atom melt ensembles used to determine the development of nanostructure and rheological properties shown in Figs 4, 5. **(D)** MD alkali-alkali partial pair distribution functions *ρ*(*r*) for the 12,096 atom glass, revealing ordering extending over three alkali coordination shells.

**Figure 2:**
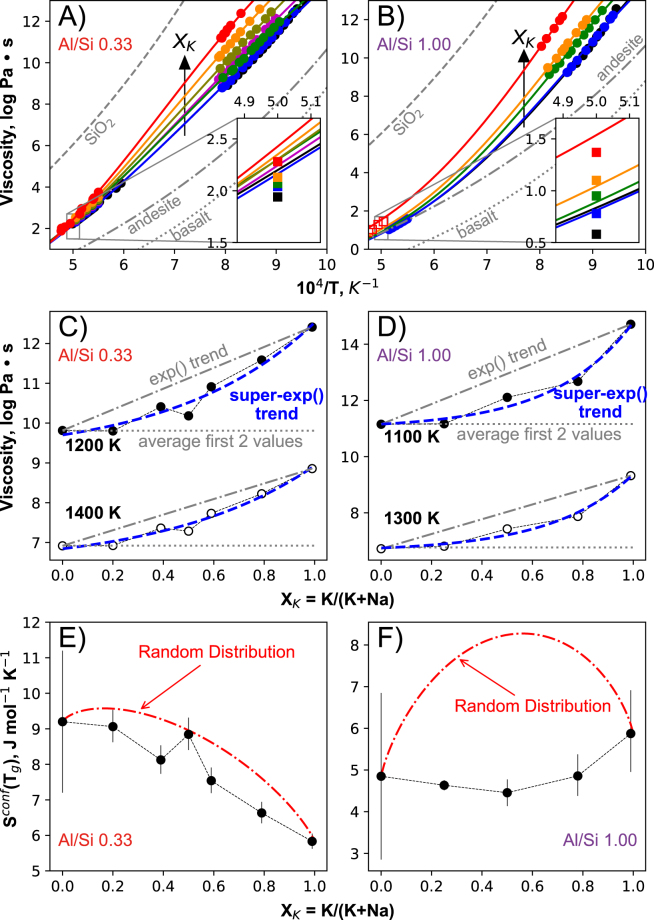
Melt viscosity and configurational entropy. (**A**,**B**) Viscosity as a function of the inverse of temperature of the melts with Al/Si = 0.33 and 1.00, as well as of reference SiO_2_^40^, andesite^41^ and basalt^42^ melts. (**C**,**D**) Iso-temperature super-exponential variations of the viscosity of the melts with Al/Si = 0.33 and 1.00 as a function of X_K_. (**E**,**F**) Variations of the configurational entropy at the glass transition temperature *T*_*g*_ of the melts with Al/Si = 0.33 and 1.0 reported as a function of *X*_*K*_. In (**A**) and (**B**), circles are measurements and lines are fits with the Adam-Gibbs model (see Supplementary Information and Table S4). In (**A**), black, blue, magenta, green, olive, orange and red symbols/curves for *X*_*K*_ = 0.00, 0.20, 0.39, 0.50, 0.59, 0.79, 0.99 respectively. In (**B**), black, blue, green, orange and red symbols/curves for *X*_*K*_ = 0.00, 0.25, 0.50, 0.78 and 1.00, respectively. Inserts show comparison of the Adam-Gibbs fits (lines) with MD simulations values at 2000 K (solid squares; see also Fig. 5). The standard deviations between the MD and Adam-Gibbs values at 2000 K are equal to 0.22 and 0.16 log units for melts with Al/Si = 0.33 and 1.0, respectively. In (**B**), the empty red squares refer to bias-corrected literature data for the glass with *X*_*K*_ = 1.0, see Supplementary Information for details. In (**C**) and (**D**), the grey dotted line highlight the initial mean viscosity values for melts with *X*_*K*_ ~ 0.0 and ~0.25, the dashed-dotted grey lines show exponential trends linking the sodic and potassic endmembers, the thick dashed blue lines highlight the super-exponential *super-exp*() trends that the data are describing, and the thin dotted dark lines between points are guide to the eyes. In (**E**) and (**F**), the red dashed lines represent variations predicted from a random mixing of Na^+^ and K^+^. Errors are given at the 1σ confidence interval; if not visible, they are smaller than symbols.

Recently, super-exponential viscosity variations were observed when mixing Na^+^ and K^+^ in compensated alumino-silicate melts rich in silica^14^, pointing to the possible clustering of alkalis. Encouraged by this, we have investigated compensated alumino-silicate melts and glasses with a broad range of silica contents (0.11 < Al/Si < 1.0; Figs 1 A, 2). In place of the viscosity depression observed upon alkali mixing in silicate melts^15^, viscosity of super-cooled compensated alumino-silicate melts (0.2 ≤ Al/Si ≤ 1.0) increases following a super-exponential trend covering 2 to 3 decades with increasing potassium content *X*_*K*_ = *K*_2_ *O/(K*_2_ *O* + *Na*_2_ *O*). Indeed, viscosity at isothermal conditions barely changes for *X*_*K*_ < ~ 0.25, but then gently increases with increasing *X*_*K*_ from ~0.25 to ~0.6, and significantly stiffens with further increase of *X*_*K*_ to 1 (Figs 1B and 2A–D). We further note that the super-exponential trend includes systematic outliers in the 0.4 < *X*_*K*_ < 0.6 chemical region at all Al/Si, suggesting some sort of transitional regime straddling the 50/50 mixed alkali composition. This particular mixed alkali behavior is especially visible at undercooled conditions, where the isothermal viscosity variations with *X*_*K*_ are larger than the transition between the isothermal viscosity of basalt and andesite melts (Fig. 2A,B), two important natural rock compositions commonly involved in effusive and explosive volcanic eruptions, respectively. The unusual mixed alkali behavior persists close to the crystalline melting points *T*_*M*_, albeit over a smaller range (Fig. 2).

We have used Molecular Dynamic (MD) simulations (see for details the Supplementary Information) to understand the structural and dynamic origins of these striking mixed alkali rheological properties. Figure 1C demonstrates for a 12096 atom glass model (Al/Si = 1, *X*_*K*_ = 0.75) how alkalis nano-segregate into clusters that can percolate in channels through the alumino-silicate network, these alkali nano-structures representing 3D manifestations of the CCRN model^11^. Besides, alkali-alkali pair distribution functions are characterized by local order extending to around 15 Å (Figs 1D, S5). We propose that developments in alkali cluster formation in compensated alumino-silicate melts are responsible for the anomalous substantial rise in melt viscosity when one metal ion is replaced by a larger one. Smaller 1512 atom models enabled computer-intensive predictions of diffusivity and viscosity to be made for numerous compositions.

### Synopsis of experiments and analysis

**Figure 3:**
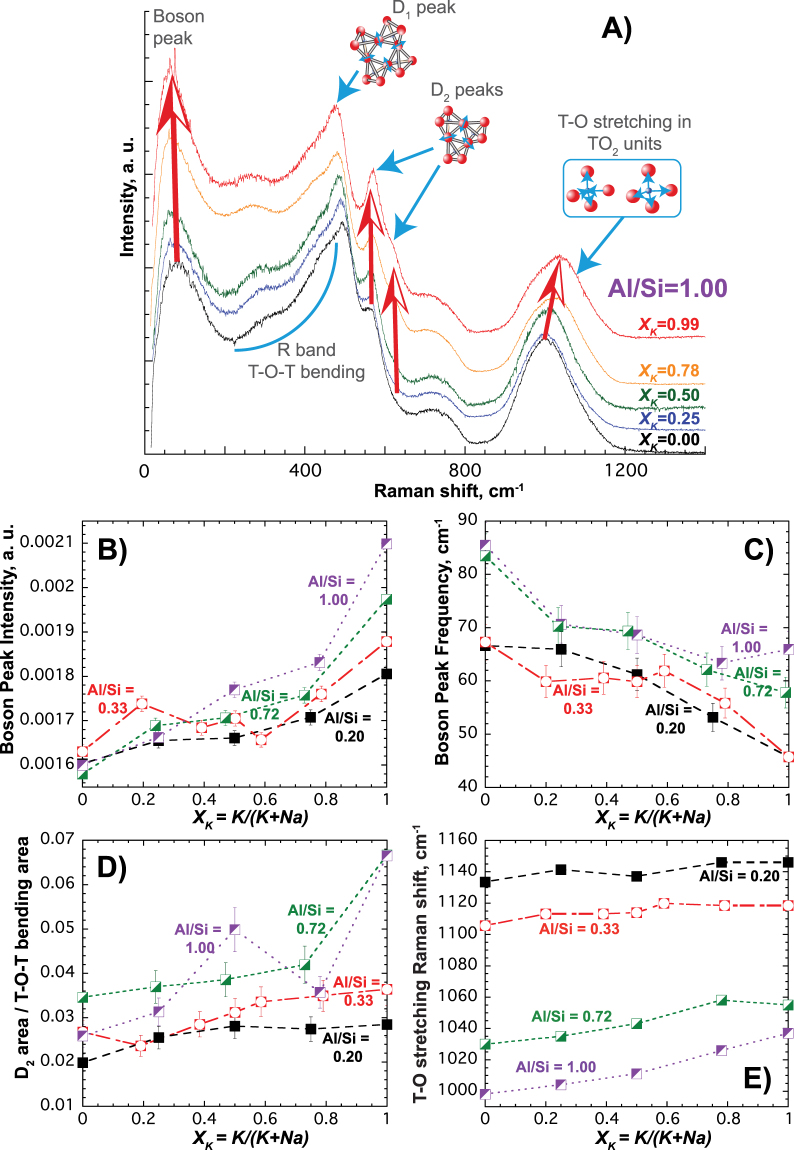
Structure of compensated glasses as shown by Raman spectroscopy. (**A**) Raman spectra of [Na_(1-*XK*)_K_*XK*_]AlSiO_4_ glasses for various MAE compositions. Arrows show the strong increases of the intensity of the Boson Peak, and of those of the *D*_1_ and *D*_2_ peaks. The band assigned to T-O (T = Si, Al) stretching in TO_4_ tetrahedral units is visible at ~1000 cm^−1^, it increases in frequency with *X*_*K*_. The R peak is assigned to T-O-T bending, and the peak at ~780 cm^−1^ to rocking of TO_4_ units. See ref.^14^ for references for those assignments. (**B**,**C**) Boson Peak Intensity and Frequency for glasses with different Al/Si ratios as a function of their *X*_*K*_. (**D**) Area of the *D*_2_ band (540–680 cm^−1^) assigned to three-membered rings normalized to the area of the total T-O-T bending modes region (230–680 cm^−1^). (**E**) Raman shift of the band assigned to T-O stretching in TO_4_ units. Errors are given at the 1σ confidence interval; if not visible, they are smaller than symbols.

**Figure 4:**
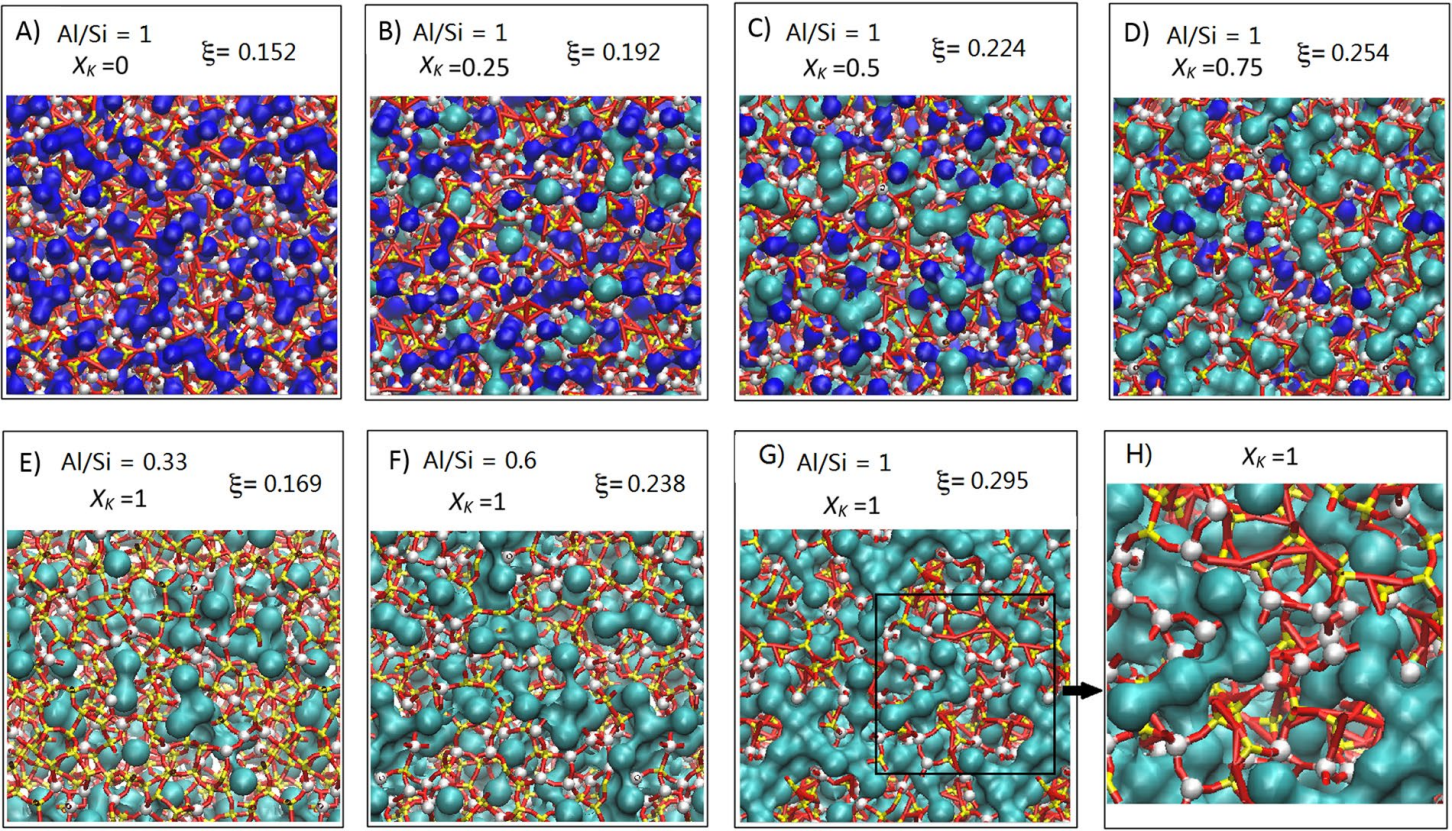
Alkali channels in mixed alkali CCRN Molecular Dynamics model of compensated alumino-silicate glasses. (**A**–**D**) Molecular graphics of the 1512 atom glass models with Al/Si = 1.0 (0 < *X*_*K*_ < 0.75). Na (blue) and K (cyan) isosurfaces are represented using respective cut-off ionic radii of 1.24 Å and 1.64 Å. Both Na and K compensating metals cluster in the glasses, but percolation pathways are visible for *X*_*K*_ > ~0.6 (**D**). See Fig. S6 for snapshots at T ~ *T*_*g*_ (1100 K). (**E**–**G**) Molecular graphics of Al/Si = 0.33, 0.66, 1 and *X*_*K*_ = 1.0 glass models, showing development of alkali clustering. (**H**) Enlargement of (**G**), illustrating Al-Al violation of Lowenstein’s Rule (Fig. 5B): Al (pink-white), Si (yellow), O (red). Melt ensemble sizes of 1512 atoms were also chosen to enable the extensive dynamics of network ions to be determined for all 15 melt models over 50 ns runs (Fig. 5F,G).

**Figure 5:**
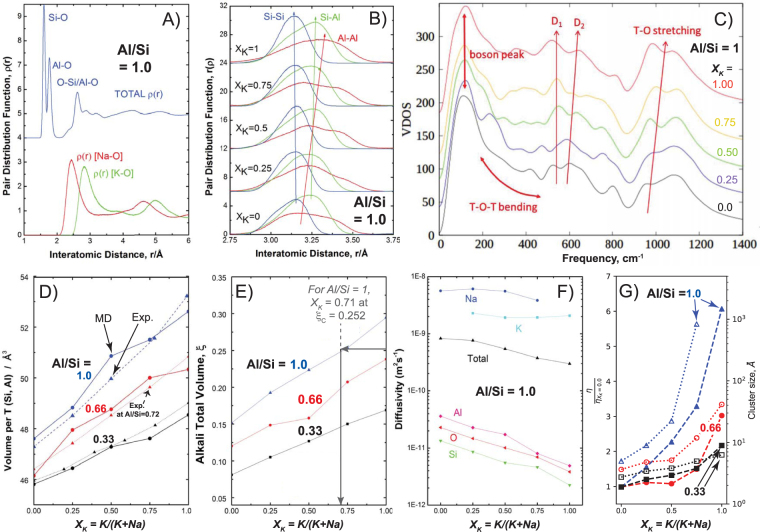
Molecular dynamics simulation results for mixed alkali compensated alumino-silicate glasses and melts. (**A**) Total and partial pair distribution functions *ρ*(*r*) of glasses at 300 K, highlighting Si-O, Al-O, Na-O and K-O in [Na_0.5_K_0.5_]AlSiO_4_ glass. (**B**) *ρ*(*r*) for network cations for Al/Si = 1 glasses with different *X*_*K*_, showing inter-tetrahedral distances increasing with *X*_*K*_ between 3.0 and 3.5 Å, 18 % Al-Al correlations averaged over all TO_4_ units. (**C**) Vibrational Density of States (Supplementary Information Methods) calculated for Al/Si = 1 glasses with *X*_*K*_ = 0.0, 0.25, 0.75, 1, increasing vertically and replicating features and trends found in Raman spectra (Fig. 3). (**D**) Experimental Molar Volumes per TO_4_ tetrahedron as a function of *X*_*K*_ for glasses with Al/Si = 0.33, 0.72 and 1, in excellent agreement with MD predictions. (**E**) Alkali total volume as a function of the non-network volume *ξ* (Supplementary Information), including the percolation threshold at *ξ*_*C*_ = 0.25. *ξ*_*C*_ is reached at *X*_*K*_ ~ 0.7 for Al/Si = 1, and at *X*_*K*_ ~ 1 for Al/Si = 0.66. (**F**) Elemental diffusivities *D*_*i*_ from MD calculations for all 15 melts at 2000 K obtained from 50 ns MSD calculations (Supplementary Information). (**G**) Cluster sizes, determined from alkali volume fractions shown in Fig. 5E, represented as a function of *X*_*K*_ together with the viscosities *η* at 2000 K (calculated from the *D*_*i*_ values, see Supplementary Information Fig. S7) for 15 different melts (Al/Si = 0.33, 0.6 and 1; *X*_*K*_ = 0, 0.25, 0.5, 0.75). Viscosities are normalized to the viscosity for *X*_*K*_ = 0. Variations of viscosity and cluster size are clearly correlated, both presenting super-exponential behavior.

**Figure 6:**
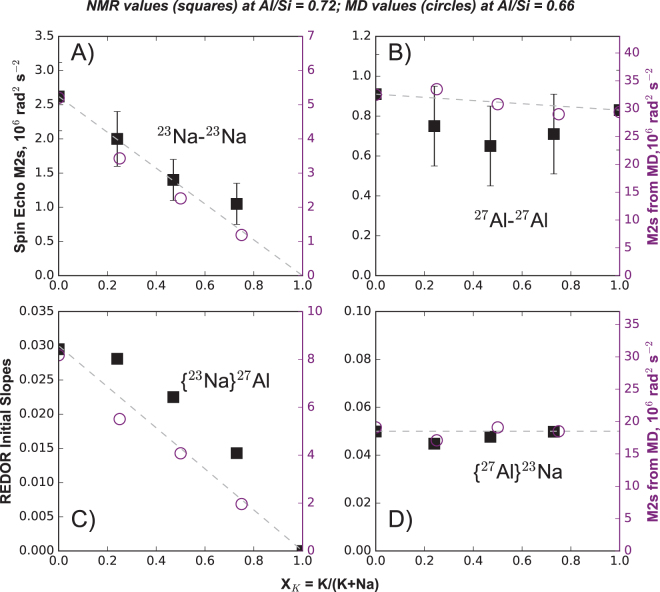
Nuclear Magnetic Resonance spectroscopy probing sodium and aluminum environments between 1.0 to 1.5 nm in alumino-silicate glasses with Al/Si = 0.72 and different *X*_*K*_. Experimental results are shown with black squares and predictions from MD simulations (Supplementary Information) for glasses with Al/Si = 0.66 are shown with open circles for comparison. (**A**) Second moments *M*_2_ determined using ^23^Na-^23^Na Spin Echo NMR spectroscopy, reflecting Na-Na distributions. (**B**) Second moments *M*_2_ determined with using ^27^Al-^27^Al Spin Echo NMR spectroscopy, reflecting Al-Al distributions. In (**C**) and (**D**), the initial slopes of the *ΔS/S*_*o*_ = *f* (dipolar evolution time) relationships observed in {^23^Na}^27^Al REDOR MAS 20 kHz and {^27^Al}^23^Na REDOR MAS 10 kHz spectra are shown. They reflect, respectively, Na distributions around Al and Al distributions around Na. See Supplementary Information for further details. Errors are given at the 1σ confidence interval; if not visible, they are smaller than symbols.

To understand how the distinctive nano-structure of alumino-silicates (Fig. 1C) relates to the dramatically non-linear mixed alkali viscosity properties (Fig. 1B), we consider first the configurational entropy *S* ^*conf*^ inherent in the viscosity values (Fig. 2). The accompanying and significant changes that occur in Raman spectra of glasses, representing the structure of melts at their glass transition, are then described, together with their attributions in modifications to the local atomic structure (Fig. 3). We go on to show, through MD simulations, how the alkali nano-structures evolve as alkalis are mixed (Fig. 4) and as the concentration of aluminum is altered. We then look in detail at pair distribution functions in melts to quantify Al-Al contacts, the extent of alkali clustering with respect to the percolation threshold, and, from anionic diffusivities, how the viscosity develops with mixed alkali composition (Fig. 5). The vibrational properties are also predicted. Finally, changes in sodium and aluminum distributions among mixed alkali compositions are explored using Spin Echo and Rotational Echo DOuble Resonance (REDOR) Nuclear Magnetic Resonance (NMR) spectroscopy, and compared with MD predictions (Fig. 6).

### Modeling non-linear viscosity behavior of alumino-silicate melts

We start by modeling the viscosity of molten alkali alumino-silicates (Fig. 2) with the Adam and Gibbs formalism (Supplementary Information) in order to estimate the configurational entropy of the melts at their glass transition temperature *T*_*g*_, *S* ^*conf*^ *(T*_*g*_ *)* ^15^. *S* ^*conf*^ *(T*_*g*_) is a thermodynamic metric of disorder in the melt at the iso-viscous condition and characterizes the residual entropy in the glassy state. It declines with increasing *X*_*K*_ in melts with Al/Si = 0.33 (Fig. 2E), and is almost constant in melts with Al/Si = 1.00 (Fig. 2F). Taking the *S* ^*conf*^ *(T*_*g*_) for molten silica of 5.1 J mol^−1^ K^−1^ ± 2.0 J mol^−1^ K^−1^ as a reference^15^, the level of order in the alumino-silicate glass structure approaches that of silica with increasing *X*_*K*_ at Al/Si = 0.33, whereas at Al/Si = 1, it actually is comparable to that of silica for any *X*_*K*_ – despite the more complex chemistry. In particular, neither glass system exhibits the parabolic excess entropy maxima expected from ideal random mixing of Na^+^-K^+^^15^. Both glass systems exhibit *S* ^*conf*^ *(T*_*g*_) variations consistent with non-random mixing of alkali metals presenting different local environments, as borne out by MD simulations (Figs 5A, S5).

### Dynamics and structure from Raman Spectroscopy of mixed alkali alumino-silicate glasses

Raman spectra of the glasses offer a vibrational point of view on the molecular landscape in the melts at *T*_*g*_. Significant changes are observed when potassium replaces sodium, as visible in Fig. 3A for Al/Si = 1 glasses (see also Figs S1 and S2 for Al/Si = 0.72 glasses). At the lowest frequencies, the intensity of the boson peak (BP) increases with increased *X*_*K*_, and its frequency decreases (Fig. 3B,C). These trends become more exaggerated for higher Al/Si ratios and associated increased compensating alkali concentrations. The BP can be assigned to collective transverse acoustic vibrational modes^16^, which in silicate glasses are promoted by cooperative inter-tetrahedral librations^17,18^. In particular, increases in BP intensity and falls in BP frequency follow decreases in glass density^18,19^ (see also Supplementary Information), and point to the size of tetrahedral rings and cages enlarging as K^+^ replaces Na^+^^19^.

The extent of nano-structural regions can be estimated from the boson wavelength *λ*_*BP*_ of cooperative vibrations^6^. Taking a typical BP frequency *ν*_*BP*_ for alumino-silicate glasses of ~70 cm^−1^ (2.1 THz; Fig. 3C) and a transverse speed of sound *v*_*t*_ in silicate glasses of ~3055 m s^−1^ ^18^, *λ*_*BP*_ = *v*_*t*_ */ν*_*BP*_ ~ 15 Å, which is around the extent of clusters visible in the MD snapshots (Figs 1 C, 4 and 5G) and of alkali-alkali correlations (Figs 1D, S5). These changes in nanoscale structure and low frequency collective dynamics, as alkali are mixed, are coupled with intensity increases in the 200–470 cm^−1^ frequency range where signals are assigned to breathing vibrations in five- and higher-membered tetrahedral rings (Figs 3A, S1). Moreover, as larger units lead to larger inter-tetrahedral angles T-O-T (with T = Si, Al)^14,20^, the frequency of the band assigned to T-O stretching vibrations in TO_4_ units should also increase with *X*_*K*_, as is observed (Figs 3A,E, S1).

As K^+^ substitutes for Na^+^, the formation of larger cohesive tetrahedral units is accompanied by increased intensity of the *D*_2_ peak visible at ~570–610 cm^−1^ (Figs 3A and D, S1) which is assigned to breathing vibrations of three-membered rings^21^. By comparison, the *D*_1_ signal, assigned to breathing vibrations of four-membered rings, is less affected. Similar *D*_2_ behavior has also been observed with substitution of Rb by Cs in compensated glasses, leading to infer that three-membered rings may be needed topologically to connect larger cohesive units^22^. In the present context, the *D*_2_ signal comprises two contributions at ~570 cm^−1^ and ~600 cm^−1^ (Figs 3A, S2), respectively assigned to rings with two SiO_4_ and one AlO_4_ units and to rings with three SiO_4_ units^21^ (see Supplementary Information and Fig. S2). Both contributions increase significantly with increasing *X*_*K*_, particularly those containing Al^3+^ cations – this is following the density decrease and associated dilation of ring topology. These changes in nano-structure, as K^+^ replaces Na^+^ in alumino-silicate glasses, become stronger with increased aluminum (Al/Si) content (Fig. 3). They mirror the dramatic non-linear viscosity behavior in the melts from which the glasses are derived, and are consistent with the predictions of MD simulations that depict clustering of compensating alkalis (Figs 1 C,4 and 5C).

Any changes in nano-segregation with increasing alkali ionic radius in compensated alumino-silicate glasses most probably relate to charge-compensation of AlO_4_^−^ tetrahedra. Notably the field strength, *Z*/*r* ^2^, of K^+^ is much lower than that of Na ^+^, with K-O bonds (*r* ~ 3.0 Å) being longer than Na-O bonds^23,24^ (*r* ~ 2.6 Å; Fig. 5A). Because of these differences, K^+^ will be less effective than Na^+^ at charge compensating individual AlO_4_^−^ in the short range. Therefore, to facilitate energy minimization, enrichment in K^+^ should lead to some increase of the medium range order around AlO_4_^−^ units. If Na^+^ and K^+^ occupy different short and medium range environments in compensated glasses as the CCRN model supports (Fig. 1D), this offers clues as to understanding the non-ideal mixing evident in viscosity data of the melts from which these glasses are quenched (Figs 1B and 2).

### Visualization of alkali cluster nano-structure in CCRNs

The alkali nano-segregation illustrated for the 12,096 model with Al/Si = 1 and *X*_*K*_ = 0.75 (Fig. 1C) is reproduced in the smaller 1512 models shown in Fig. 4. These show the evolution of alkali nano-segregation as potassium replaces sodium. Where clustering of sodium is obvious when *X*_*K*_ is low, elsewhere these nano-structural units enlarge with potassium content, becoming continuous for *X*_*K*_ = 0.75 and 1, where the viscosity increases dramatically (Figs 1B and 2D). It is also noticeable that alkali clusters associate with aluminum-rich network regions (Fig. 4H), in violation of Lowenstein’s Rule for crystalline alumino-silicates^25^. As the aluminum content is reduced from Al/Si = 1.0 to 0.33, and with it the concentration of compensating alkalis, the nano-clustering reduces progressively alongside aluminum clustering (Fig. 4E–G).

### Glass atomic structure and dynamics of CCRNs

The partial pair distribution functions predicted from MD simulations for glasses with Al/Si = 1 shows the distinction in the network between SiO_4_ and AlO_4_^−^ tetrahedra, as well as the distinct immediate environments of the compensating alkalis in the glass (Fig. 5A). Strong alkali-alkali correlations illustrated in Fig. 1D are present for all compositions (Fig. S5) and specify the alkali clustering evident in the molecular graphics (Figs 4, S6). In addition to the regular inter-tetrahedral Si-Si and Si-Al correlations, there is a significant fraction of Al-Al correlations amounting to ~18 % of all the inter-tetrahedral distances (Fig. 5B). This is comparable to the experimental result of 13 % reported from ^17^O MAS NMR^26^. Each is less than the 25 % expected if aluminum was randomly distributed throughout the network, and therefore consistent with the clustering of aluminum observed with MD in Fig. 4H (Supplementary Information Methods). Moreover, Al-Al and Si-Al distances increase when K^+^ replaces Na^+^ (Fig. 5B), pointing to an increase in the mean inter-tetrahedral T-O-T angle, thus corroborating interpretations of the frequency increase of the Raman T-O stretching band (Fig. 3E). All the major features in the Raman spectra (Fig. 3A) are reproduced in the computed Vibrational Density of States (VDOS) for the Al/Si = 1.0 glass series (Fig. 5C).

### Predicting alkali percolation, melt diffusivity and viscosities

The glass molar volume per tetrahedron, determined from glass densities (Table S1), increases linearly as potassium replaces sodium in all the glasses, a behavior accurately predicted by MD calculations (Fig. 5D). The volume occupied by Si and Al cations remains approximately fixed (Supplementary Information Methods), so rises in molar volume with *X*_*K*_ reflect the increasing number of large K^+^ ions replacing smaller Na^+^ ions. At low *X*_*K*_, both cations cluster in the tetrahedral network (Fig. 4A–C). As *X*_*K*_ increases further, percolation thresholds occur for Al/Si = 1 (Fig. 4D,G) and for Al/Si = 0.66 (Fig. 4F). Approximating alkalis as randomized octahedra, the percolation threshold is predicted to occur whenever the alkali total volume *ξ* exceeds 0.252^27^. For sodium clusters in NaAlSiO_4_, *ξ* = 0.151 < *ξ*_*C*_, explaining why percolation is not observed at low *X*_*K*_ values in the Al/Si = 1.0 glasses (Fig. 4A). In comparison, for potassium clusters in KAlSiO_4_, *ξ* = 0. 295 > *ξ*_*C*_, explaining why percolation channels are observed at high *X*_*K*_ values in this glass series (Fig. 4D,G). With mixing Na^+^ and K^+^, the total alkali volume *ξ* becomes higher than *ξ*_*C*_ for *X*_*K*_ > ~ 0.71 for Al/Si = 1 glasses (Fig. 5E). Likewise, for Al/Si = 0.66 glasses, *ξ*_*C*_ aligns with *X*_*K*_ > ~ 0.9. For Al/Si = 0.33 glasses, *ξ*_*C*_ is never reached as potassium replaces sodium. Therefore, percolation of alkali channels should occur in glasses rich in Al^3+^ and K^+^ (Fig. 4D,G) while only alkali clusters are expected in glasses rich in silica (Fig. 4E,F). The occurrence of percolation explains the strong super-exponential trend observed in viscosity data as well as concomitant increases in BP intensity and* D*_2_ ring fraction occurring as *X*_*K*_ reaches ~0.7 for compositions with Al/Si = 1.0 and 0.72 (Figs 1B, 2D and 3). For compositions with Al/Si = 0.33 and 0.2, the absence of percolation of Na^+^ and K^+^ clusters in the melts explains the fact that the super-exponential trend in isothermal viscosity versus *X*_*k*_ plots (Figs 1B, 2C) is slightly less evident than for melts with higher Al/Si where the percolation threshold can be reached. In all cases, increasing viscosity with increasing K^+^ fraction is assigned to the lower field strength of K^+^ compared to Na^+^, the larger K^+^ promulgating the dilation of the network, evident in Raman experiments and MD calculations (Figs 3, 5B), this resulting in a strengthening of Al-O and Si-O bonds as they shorten^28^.

The T-O bond strengthening as K^+^ replaces Na^+^ in the melts and glasses is further evident from the decreasing simulated diffusivities *D*_*i*_ of the network cations, shown in Fig. 5F at 2000 K for the Al/Si = 1.0 melts. *D*_*i*_ were obtained over 50 ns to encompass the diffusive regime of all ions in the melts (Supplementary Information Methods). The simulated *D*_*i*_ at 2000 K lead to melt viscosities in excellent agreement with experimental values (Figs 2A,B, S7). The MD results confirm that the alumino-silicate network stiffens as K^+^ replaces Na^+^ as the compensator ionic field strength decreases (Fig. 5F), yielding *η* principally governed by the Si, Al and O mobilities (Fig. S7) and increasing non-linearly with *X*_*K*_ (Fig. 5G). This effect increases with the melt alkali and Al^3+^ content at super-liquidus temperatures (Fig. 5G) and is even more evident at super-cooled temperatures, close to *T*_*g*_ (Fig. 1B). Overall, observations and simulations point to the central role played by alkali nano-structures (Figs 1, 4 and 5) on the properties of alumino-silicate systems.

Using the MD simulations, the super-exponential behavior of the melt viscosity with *X*_*K*_ observed experimentally (Figs 1B and 2C,D) can be linked with increased alkali nano-cluster sizes as the percolation threshold is reached (Supplementary Information Methods). Notably, the percolation transition has a width $|p\,-\,{p}_{c}|$, where *p* is the probability of conduction within a cluster of length *l* atomic shells and *p*_*c*_ the value at the percolation transition where *l* → ∞ on macroscopic scales. Importantly, the percolation width is inversely related to the cluster length *viz*. $|p-{p}_{c}|={p}_{c}/l$ ^29^. Given that *p* ∝ *ξ* and therefore that $|\xi -{\xi }_{c}|={\xi }_{c}/l$, we take values of the alkali fraction *ξ* from Fig. 5E to obtain the changing cluster size with potassium content *X*_*K*_ for different Al/Si compositions as shown in Fig. 5G (see also Fig. S8). Comparing this with the predicted melt viscosities in Fig. 5G, they share the same super-exponential character, notably for Al/Si = 0.66 and 1. This indicates that, as alkali clusters grow in size, *l*.*r*_*M-M*_, towards the bulk percolation transition (Fig. 4), they will increasingly immobilize the liquid network leading to similar super exponential growth in viscosity (Fig. 5G), as found experimentally (Fig. 2C,D).

### Testing MD predictions with Spin Echo and REDOR NMR

Turning finally to NMR experiments, the picture emerging so far linking nano-structure with melt properties is extended to exploring spatial proximities from through-space dipolar interactions, which can be directly evaluated from the structures predicted by MD CCRN simulations (Fig. 6). We have carried out two types of experiments: measurement of homonuclear X-X dipolar couplings from spin-echo experiments^30^ and measurement of heteronuclear X-Y dipolar couplings from REDOR experiments^31,32^. Both techniques translate in the measurement of dipolar coupling, through second moment M2 or REDOR *ΔS/S*_0_ initial slopes, that are proportional to the sum of *1/r* ^6^ (*r* being the internuclear distances). These have a field of view of 10 to 15 Å around the observed nuclei, usually considered to equate with long range order^33^ and lying well within the box size (30 Å) of the MD calculations. Results of available NMR experiments for the Al/Si = 0.72 glass series are compared with MD predictions (eqn S7) for glasses with Al/Si = 0.66 in Fig. 6. The small difference in Al/Si does not radically affect the structure of the glass series, as confirmed by the good overall agreement between NMR and MD data. Starting with homonuclear data, the ^23^Na-^23^Na M2 Spin Echo generally deceases as K^+^ replaces Na^+^, but begins to level out beyond *X*_*K*_ = 0.5 (Fig. 6A), pointing to increased agglomeration of Na^+^ in the long range, and, by implication, of K elsewhere. These *X*_*K*_ values coincide with sharp increases in viscosity (Figs 1B and 5G), in Raman BP and *D*_2_ intensity (Fig. 3) as well as in the size of alkali clusters (Fig. 5G) in melts and glasses with Al/Si ≥ 0.6. By contrast, there is a small minimum at *X*_*K*_ = 0.5 for the ^27^Al-^27^Al Spin Echo (Fig. 6B) with a small decrease overall. Considering the Al^3+^ content as constant, this observation is consistent with a change in nano-structural Al-Al correlations for these glasses in the long range. Turning to the heteronuclear data, the almost linear trend in MD M2 for {^23^Na}^27^Al suggests random positioning of Na^+^ around Al^3+^ within 10–15 Å for the CCRN (Fig. 6C). {^23^Na}^27^Al REDOR NMR values are similar, if a little higher, potentially pointing to some inflation of Na^+^ clustering in the glass. Unexpectedly, the Na^+^ environment of Al^3+^ is scarcely affected by substitution of K^+^ (Fig. 6C,D). This may either reflect a preferred association of AlO_4_^−^ with Na^+^ cations because of their higher ionic field strength, and, thus, better compensating character than K^+^, or a lack of extensive Na-K mixing as evident from the variation of the configurational entropy and the MD snapshots (Figs 2E,F and 4B–D).

## Conclusions

The Molecular Dynamic simulations exemplifying the CCRN concept offer an explanation, in terms of the alkali clustering and percolation pathways in the framework of the CCRN model, of the non-linearity observed in the rheological properties of alumino-silicate melts and mirrored in dynamical experiments of glasses. Increasing the size and decreasing the ionic field strength of metal cations in compensated alumino-silicate melts results in an increase in the ordering of the molecular network at intermediate distances (Figs 3–6) because the metal cations present different local environments (Figs 1D and 5A, S5). Therefore, substitution of a small compensator by a large one serves to dilate tetrahedral rings and cages in the network, increasing the network mean inter-tetrahedral angle (Figs 3 and 5B), resulting in shortening and strengthening Si-O and Al-O bonds (Fig. 3E). Thus, diffusivities of Si, O and Al decrease (Figs 5F, S7). As cooperative movement of network ions is increasingly inhibited, viscosity rises super-exponentially (Figs 1B, 2 and 5G). Metal cations mix non-randomly in the glass structure (Fig. 2E,F), each one presenting its defined local order (Figs 1D and 5A, S5). In Na-rich melts, the melt viscosity is dominated by the properties of the Na-Al-Si-O subnetwork, with Na^+^ slightly clustering around Al^3+^ to ensure charge balance (Fig. 4A), this effect being promoted by the presence of Al-O-Al bonds in the glass network (Fig. 5B). The addition of K^+^ in the free volume of the network of Na-rich melts (*X*_*K*_ < ~0.25) induces only a mild isothermal viscosity increase (Figs 1B and 2C,D). Further increase in *X*_*K*_ leads to a transition at 0.4 < *X*_*K*_ < 0.6, marked by systematic isothermal viscosity deviations from perfect super-exponential trends reflecting changes in the diffusion of network ions (Figs 2C,D and 5F). At the same time, Na^+^ and K^+^ diffuse within clusters and channels in the melt via the energetically most advantageous environments, resulting in almost constant behaviour (Fig. 5F) because of segregation of subnetwork geometries^4,11^ (Fig. 4B–D); different packing constraints are manifest in differing medium range order (Figs 3C,E and 5A,B) which is frozen into the CCRN glass. At *X*_*K*_ > ~ 0.6, the viscosity significantly stiffens as the melt properties are mostly governed by those of network anions in the K-Al-Si-O subnetwork. The latter is particularly viscous as K^+^ tends to cluster in specific, large cationic sites (Fig. 5A), promoting Al-O-Al linkages (Fig. 5B), separating the network regions. Those effects result in an extended clustering and even channeling of K^+^ in the structure of compensated melts (Fig. 4E–H), yielding few possibilities for network re-arrangement, immobilizing the network liquid leading to declining anion diffusion and non-linear increases in viscosity (Fig. 5G). In mixed Na-K melts, percolation of clusters in channels occurs at high Al/Si and high *X*_*K*_, the width of the percolation transition extending to lower *X*_*K*_ with non-linear changes in cluster sizes (Fig. S8), further reinforcing the super-exponential viscosity increase following *X*_*K*_ increase (Fig. 5G). Alkali clusters, channels and contortions of the network are transitional, dynamic features, this time-dependence making them different from a steady phase separation at the nanometre scale common in modified silicates. In peralkaline glasses, where metal cations concentration exceeds that needed for aluminum charge compensation, we envisage a combination of CCRN and MRN models, where metallic clustering occurs in both silicate and alumino-silicate regions.

This study has shown how the concentration and size of charge compensator cations controls the extent of alkali nano-structures, large compensating cations clustering and percolating through the tetrahedral network more easily than small ones. In this regard, we would expect alumino-silicate glasses containing compensating Rb^+^ or Cs^+^ to behave in a similar manner, but glasses containing smaller compensating alkalis like Li^+^ would be less likely to exhibit a percolation threshold. Such field strength control may also exist for network modifier cations in silicates composition, but the outcome for ionic transport and rheology will be dissimilar, because charge compensator and network modifier cations differently affect the mobility of network ions that dominate the melt viscosity. For instance, while network modifiers like Li^+^, Na^+^ and K^+^ segregate in silicate glasses, Li^+^ cations seem to cluster more than larger alkalis with smaller inter-channels distances^5,7,8,33^. This probably explains the strong tendency of Li_2_O-SiO_2_ melts to macroscopically phase separate close to *T*_*g*_^34^. Therefore, the ionic radius of metal cations in oxide glasses influences the formation of clusters and percolation of channels inside the tetrahedral network, but in ways depending on whether these act as network modifiers or as charge compensators, each one being respectively described by the MRN and CCRN models^3,6,11^. In general, percolation channels seem to be universal features in the structure of silicate and alumino-silicate melts and glasses quenched from them.

Because channels establish preferential pathways for diffusion of non-network former elements^6,11^, they will influence the processes stemming from ionic transport, such as nucleation and crystallization, volatile degassing, and Redox processes. This is shown, for instance, by the channelization of Zr^4+^, a well-known nucleating agent in melts, that proceeds melt crystallization^35^. Similar observations have been made for Mo^6+^, an element present in highly radioactive liquid wastes produced during spent nuclear fuel reprocessing, and immobilized in borosilicate glass matrices^36^. Turning to volatile elements, percolation channels have further been proposed as key features for the diffusivity of noble gas in magmas^37^. Thus, recognizing such nano-structural amorphous heterogeneities, from the supercooled state to the quenched glass, could well be the key to solving many Earth, Material sciences and Industrial problems.

## Methods

### Starting Materials

Compensated glasses with Al/Si = {0.20, 0.33, 0.72, 1.00} and 0.0 ≤ *X*_*K*_ = K_2_O/(K_2_O + Na_2_O) ≤ 1.0 were prepared following the protocol described in Le Losq and Neuville^14^. Densities of all samples have been measured with the Archimedes method using toluene as the immersion liquid (Table S1). Chemical compositions have been measured using a Cameca SX50 electron microprobe at the CAMPARIS facility of the University Paris VI (France), with a 30 nA current, U = 30 kV, and 5 s of counting. Chemical compositions are the mean of 10–20 individual measurements. All glasses are uncolored and transparent, and no crystallization has been detected by optical microscope, Raman spectroscopy or in the electron diffraction pattern during HRTEM observations.

### Viscosity and Analysis

Viscosity measurements were performed with a creep-apparatus following the method described in Le Losq and Neuville^14^. All samples were transparent after experiments, indicating that no crystallization occurred. No difference of density measured before and after experiments was observed and Raman spectra of deformed samples are identical to those of the initial glasses, confirming the absence of crystallization. The viscosity of all samples was Newtonian within the range of applied stresses in the creep experiments. Measured viscosity are provided in Tables S2 and S3 in Supplementary Information. When possible, the data were fitted using the equation proposed by Richet^15^ and based on the Adam-Gibbs theory of liquid relaxation^38^. Details are provided in the Supplementary Information, and Adam-Gibbs modelling parameters in Table S4.

### Raman and NMR Spectroscopy

Raman spectra were recorded using a T64000 Jobin-Yvon^®^ Raman spectrometer equipped with a confocal system, a 1024 charge-couple detector cooled by liquid nitrogen and an Olympus^®^ microscope. The optimal spatial resolution allowed by the confocal system is 1–2 μm^2^ with a ×100 Olympus^®^ objective, and the spectral resolution is 0.7 cm^−1^. A Coherent^®^ laser 70-C5 Ar^+^, having a wavelength of 514.532 nm, has been used as the excitation line. Unpolarized Raman spectra were acquired between 20 and 1500 cm^−1^ on pieces of glass from the starting materials that were excited with a laser power of 200 to 250 mW on the sample. Unprocessed Raman spectra are provided in a spreadsheet in  supplementary Dataset 1.

The REDOR {^23^Na}^27^Al and {^27^Al}^23^Na NMR as well as ^23^Na-^23^Na and ^27^Al-^27^Al spin echo experiments have been obtained on a Bruker Avance III 17.6 T spectrometer operating at frequencies of 195.5 MHz and 198.4 MHz respectively, using radio-frequency fields of 10.0 kHz and 12.5 kHz respectively (measured selective π/2 pulse duration of 8.5µs and 10.0µs). The REDOR experiments were performed spinning at 20 kHz while Spin Echo experiments where performed under static conditions, at −50 °C, with the sample located in the middle of the 4 mm rotor. During all experiments a duplexer (NMR Service GmbH) was used to double-tune a single channel of the NMR probe to the two very close ^27^Al and ^23^Na frequencies.

### Molecular Dynamic Simulations

DLPOLY classic^39^ was used to perform MD simulations to create the 12,096 atom CCRN model for Al/Si = 1 glass shown in Fig. 1. DLPOLY classic was also used to generate the 1512 atom models of melts shown in Fig. 4, and the mixed alkali melt models with Al/Si = 0.33, 0.66, 1, whose viscosities were predicted from 50 ns simulations (Fig. 5). Full details of these 15 CCRN melts and glasses used in this study are given in the Supplementary Information.

### Data availability

Data generated or analysed during this study are either directly included in this published article and its Supplementary Information files or available from the corresponding author on request.

## Electronic supplementary material

SupplementaryInformation

Supplementary Dataset 1
