# Supplementary material for: Percolation channels: a universal idea to describe the atomic structure and dynamics of glasses and melts
Source: Sci Rep. 2017 Nov 28;7:16490. doi: 10.1038/s41598-017-16741-3 (PMC5705680; doi:10.1038/s41598-017-16741-3)
Supplement: Supplementary file 1 — SupplementaryInformation [file 41598_2017_16741_MOESM1_ESM.pdf]

Percolation channels: a universal idea to describe the atomic structure and dynamics of glasses and melts

Charles Le Losq[1,2*], Daniel R. Neuville[1], Wenlin Chen[3], Pierre Florian[4], Dominique Massiot[4], Zhongfu Zhou[3], George N. Greaves[3,5,6]

Supplementary Information

Supplementary Methods

Chemical composition of the samples

Table S1: Chemical compositions measured in wt% (measurements) and mol% (calculated from wt%), relative density and glass transition temperature T_g of the Na-K compensated glasses listed in Fig. 1A. Compositions with Al/Si of 0.20 and 0.33 can be retrieved in Le Losq and Neuville[14].

Al/Si	X_K	wt% SiO_2	wt% Al_2O_3	wt% K_2O	wt% Na_2O	mol% SiO_2	mol% Al_2O_3	mol% K_2O	mol% Na_2O	relative density	T_g, K
**0.11**	**1.00**	83.74(24)	07.90(15)	07.26(13)	00.00(3)	*90.02*	*05.00*	*04.98*	*00.00*	n.m.	n.m.
**0.50**	**0.00**	59.41(45)	25.46(31)	00.02(2)	15.11(45)	66.70	16.84	00.01	16.44	2.422(6)	1091.5
**0.50**	**0.99**	54.21(38)	23.62(18)	21.08(15)	00.10(2)	*66.37*	*17.04*	*16.46*	*00.12*	2.401(5)	n.m.
**0.72**	**0.00**	49.96(93)	31.40(52)	00.00(2)	18.71(37)	*57.69*	*21.37*	*00.00*	*20.94*	2.465(1)	1072.8
**0.72**	**0.24**	49.24(26)	30.49(22)	06.25(5)	13.33(10)	*58.54*	*21.36*	*04.74*	*15.36*	2.462(3)	1085.8
**0.72**	**0.47**	48.13(46)	29.82(15)	12.33(12)	09.05(19)	*58.45*	*21.34*	*09.55*	*10.66*	2.460(4)	1103.9
**0.72**	**0.73**	47.87(41)	29.64(23)	17.71(13)	04.23(08)	*59.29*	*21.64*	*13.99*	*05.08*	2.452(2)	1144.6
**0.72**	**0.99**	46.41(54)	28.40(41)	24.94(52)	00.13(04)	*58.61*	*21.14*	*20.09*	*00.16*	2.442(2)	1249.3
**1.00**	**0.00**	41.59(40)	36.12(55)	00.04(4)	21.75(46)	*49.54*	*25.35*	*00.00*	*25.11*	2.494(2)	1073.8
**1.00**	**0.25**	40.99(40)	35.00(33)	07.94(26)	16.06(28)	*49.84*	*25.08*	*00.16*	*18.93*	2.499(5)	1072.5
**1.00**	**0.50**	40.29(20)	33.97(35)	15.45(29)	10.28(25)	*50.28*	*24.98*	*12.30*	*12.44*	2.494(4)	1104.9
**1.00**	**0.78**	38.80(36)	33.33(26)	22.99(22)	04.32(11)	*50.20*	*25.41*	*18.97*	*05.42*	2.489(4)	1125.8
**1.00**	**0.99**	38.51(55)	32.20(43)	29.40(34)	00.10(05)	*50.45*	*24.86*	*24.57*	*00.13*	2.463(6)	1192.6

Adam and Gibbs modelling of the viscosity

The Adam and Gibbs theory of viscous flow[38] allows relating the configurational entropy of silicate melts to their viscosity through the equation[15]

$$\log(\eta) = Ae + \frac{Be}{TS^{conf}(T)}, \quad (S1)$$

with A_e a pre-exponential term equals to the viscosity at infinite temperature, B_e (J mol^{-1}) a constant proportional to the potential barrier opposed to the cooperative rearrangement of the liquid structure, $S^{conf}(T)$ (J mol^{-1} K^{-1}) the melt configurational entropy, η its viscosity (Pa · s) and T its temperature (K). $S^{conf}(T)$ can be written as equal to[15]:

$$S^{conf}(T) = S^{conf}(T_g) + \int_{T_g}^{T} Cp^{conf}(T)/T\, dt, \quad (S2)$$

with

$$Cp^{conf}(T) = Cp_l(T) - Cp_g(T_g). \quad (S3)$$

In Eqs. (S2) and (S3), $S^{conf}(T_g)$ is the configurational entropy of the melt at the glass transition temperature T_g, $Cp^{conf}(T)$ is the liquid configurational heat capacity at T, $Cp_l(T)$ is the liquid heat capacity at T, and $Cp_g(T_g)$ is the glass heat capacity at T_g. During the ideal mixing of two alkali or alkaline-earth cations in silicate melts, it is possible to model the variation of $S^{conf}(T_g)$ as the sum of a topological contribution S^{topo} and a chemical mixing contribution S^{mix} [15,43]

$$S^{topo} = \sum_i x_i S_i^{conf}, \quad (S4)$$

$$S^{mix} = -nR \sum_i x_i \ln(x_i), \quad (S5)$$

$$S^{conf}(T_g) = S^{topo} + S^{mix}, \quad (S6)$$

with S_i^{conf} the partial configurational entropy of the endmember i, x_i its concentration, n the number of atom exchanged per unit formula, and R the ideal gas constant. Using equation (S5) assumes a random mixing of the cations between the structural sites[15,43]. We tested this model for melts with Al/Si = 0.33 and 1.0, for which high temperature data and $S^{conf}(T_g)$ values are available.

At Al/Si = 0.33, high-temperature viscosity measurements[44,45] are available for the melts with $X_K = 0.0$ and 1.0. For melts with intermediate X_K values, we interpolated the high-temperature viscosity between 1820 and 1980 K (temperature range at which data for both the sodic and potassic endmembers are available), as they are expected to vary almost linearly with X_K. $Cp_g(T_g)$ has been calculated with using the model from Richet[46]. For melts with with $X_K = 0.0$ and 1.0, $Cp_l(T)$ values were determined with using the heat capacity data from Richet and Bottinga[47]. For melts with intermediate X_K values, we linearly interpolated the $Cp_l(T)$ values.

At Al/Si = 1.00, reliable viscosity and heat capacity measurements[44,47,48] are available for the melt with X_K = 0.0. Data from N'Dala et al.[49] also exist for the melts with X_K = 0.0 and 1.0. However, the values for X_K = 0.0 are shifted of ~ 0.8 log unit compared to data from Riebling[44] and Toplis et al.[48], such that they probably are poorly reliable. We thus excluded them from the fits. As the melt with X_K = 0.25 presents viscosity values nearly undistinguishable from those of the melt with X_K = 0.0 at low temperature (Fig. S3A), their high-temperature viscosity should be undistinguishable. As a consequence, we also used the high-temperature viscosity of the melt with X_K = 0.0 for constraining that of the melt with X_K = 0.25. For the other melts, no high temperature viscosity data were used during the fits. $Cp_g(T_g)$ has been calculated with using the model from Richet[46]. As no liquid heat capacity data are available for the melt with X_K = 1.0, we used the model from Richet and Bottinga[50] to calculate $Cp_l(T)$. Using this model may result in errors as high as 5% on $Cp_l(T)$. However, this should not translate in high errors on $S^{conf}(T_g)$, as this parameter is mainly influenced by the behaviour of low temperature viscosity values whereas $Cp_l(T)$ values are more coupled with viscosity variations at temperature much higher than T_g (eqn S2). This assumption is confirmed by estimating the $Cp_l(T)$ values of melts with Al/Si = 0.33: $S^{conf}(T_g)$ values determined with using experimental[47] or model-derived[50] $Cp_l(T)$ values are very close. For instance, at X_K = 1.0, we determined with using the experimentally derived $Cp_l(T)$ that $S^{conf}(T_g)$ = 5.83 J mol^{-1} K^{-1} ± 0.11 J mol^{-1} K^{-1} (Table S4). With using model-derived $Cp_l(T)$ values, we calculated $S^{conf}(T_g)$ = 6.65 J mol^{-1} K^{-1} ± 0.11 J mol^{-1} K^{-1}. Both $S^{conf}(T_g)$ values are very close and embedded in the error bar of the calorimetric $S^{conf}(T_g)$ value[15], equal to 7.1 J mol^{-1} K^{-1} ± 2 J mol^{-1} K^{-1}. Furthermore, the error introduced with using the $Cp_l(T)$ model is systematic: all $S^{conf}(T_g)$ values are shifted up of ~ 0.3-0.5 J mol^{-1} K^{-1} and the trend described in Figure 2D is unchanged.

After selection of relevant viscosity and heat capacity values, the modelling was performed with minimizing the least-square criterion between calculated and measured viscosities for each glass series with the Levenberg-Marquardt algorithm. The pre-exponential factor *Ae* in eqn (S1) was set as a common parameter for a given glass series. To improve the convergence of the minimization algorithm and the robustness of the results, the $S^{conf}(T_g)$ value for the sodic X_K = 0.0 end members were fixed to their calorimetric values during the least-square minimization (values from Richet[15], Richet and Bottinga[47], Richet et al.[51]). This was particularly important for the Al/Si = 1.00 melts, for which few high-temperature viscosity data are available. For this melt series and at X_K = 1.0, the extrapolation at high temperature of the model agrees with the viscosity data from N'Dala et al.[49] corrected from the 0.8 log unit bias discussed previously (Fig. 2B). This validates the adopted approach and highlights the ability of the Adam and Gibbs model for viscosity extrapolation.

Table S2: Viscosity (log_{10} Pa · s) and temperature (kelvin) data for melts with Al/Si = 1.0. Experimental errors are smaller than 0.03 log unit.

**Al/Si = 1.0**									
X_K = 0.0		X_K = 0.25		X_K = 0.50		X_K = 0.78		X_K = 0.99	
T, K	log η, Pa · s	T, K	log η, Pa · s	T, K	log η, Pa · s	T, K	log η, Pa · s	T, K	log η, Pa · s
1058.9	12.57	1065.6	12.23	1089.5	12.48	1125.8	12	1186.7	12.16
1072.5	12.04	1071.3	12.04	1105.7	11.99	1135.6	11.71	1194.3	11.96
1085.4	11.59	1084.1	11.62	1119.7	11.56	1151.2	11.32	1209.1	11.54
1093.2	11.33	1099.7	11.18	1132.3	11.2	1172.3	10.78	1230.6	11.01
1102.1	11.09	1110.9	10.84	1148.8	10.78	1181.9	10.5	1245.2	10.61
1110.7	10.79	1127.2	10.39	1164.0	10.37	1188.0	10.35		
1121.1	10.5	1148.4	9.87	1172.6	10.19	1202.8	9.98		
1134.3	10.12	1162.7	9.54	1193.6	9.73	1227.2	9.49		
1150.7	9.72	1174.4	9.24	1207.5	9.43				
1172.5	9.26			1222.8	9.14				

Table S3: Viscosity (log_{10} Pa · s) and temperature (kelvin) data for melts with Al/Si = 0.72. Experimental errors on viscosity are smaller than 0.03 log unit.

**Al/Si = 0.72**									
X_K = 0.0		X_K = 0.24		X_K = 0.47		X_K = 0.73		X_K = 0.99	
T, K	log η, Pa · s	T, K	log η, Pa · s	T, K	log η, Pa · s	T, K	log η, Pa · s	T, K	log η, Pa · s
1063.2	12.33	1079.3	12.19	1096.6	12.22	1135.2	12.26	1244.4	12.12
1073.3	11.97	1099.8	11.59	1101.2	12.07	1155.2	11.73	1254.7	11.87
1086.4	11.58	1113.4	11.28	1117.8	11.6	1174.3	11.25	1264.6	11.65
1094.3	11.34	1124.8	10.96	1125.3	11.42	1200.4	10.73	1268.6	11.48
1106.3	10.99	1133.7	10.69	1141.1	10.99	1215.6	10.41	1269.2	11.45
1122.8	10.56	1140.3	10.6	1152.1	10.78	1226.2	10.2	1274.1	11.37
1139.3	10.12	1154.9	10.23	1175.7	10.2	1227.2	10.17	1275.8	11.32
1154.2	9.75	1169.7	9.89	1185.8	9.98	1236.6	9.99	1278.4	11.25
1173.3	9.33	1186.4	9.56	1202.7	9.62	1247.3	9.77		
		1212.4	9.06	1217.3	9.34				
				1231.8	9.05				

Table S4: Parameters of the Adam and Gibbs viscous modelling. Errors indicated in parenthesis are given at the 1σ confidence interval. Two positive and negative values are provided in the case the error analysis returned strongly asymmetric confidence intervals. *$S^{conf}(T_g)$ values for melts with X_K = 0.00 have been fixed to their values determined from calorimetric measurements during the least-square minimization.

Al/Si	X_K	***Ae***	***Be, J mol⁻¹ K⁻¹***	$S^{conf}(T_g)$, ***J mol⁻¹ K⁻¹***
	**0.00**		146640(478)	9.17(2.0)*
	**0.20**		143710 (3660)	9.06(0.23)
	**0.39**		132940(3310)	8.12(0.20)
**0.33**	**0.50**	-2.70 ± 0.04	142420(3700)	8.84(0.23)
	**0.59**		126470(3060)	7.54(0.18)
	**0.79**		114750(2670)	6.63(0.15)
	**1.00**		104670(1970)	5.83(0.11)
	**0.00**		69844(184)	4.85(2.0)*
	**0.25**	-1.53 ± 0.03	66705(927)	4.63(0.06)
**1.00**	**0.50**		66523(2280)	4.45(0.16)
	**0.78**		73906(-3414/+3755)	4.85(-0.24/+0.26)
	**0.99**		94746(-7305/+8613)	5.87(-0.46/+0.54)

Processing of the Raman Spectra

After acquisition of the Raman spectra, a linear horizontal baseline was subtracted from the spectra. Their total areas were calculated to normalize their intensities, with a total area of unity, equated with the sum of the vibrational density of states (VDOS). BP intensity and frequency have been determined using the Matlab® software, smoothing slightly the signal to avoid measuring small spikes. After doing that, the Matlab® code was adapted in order to constrain a polynomial baseline to fit the base of the D_2 peak. With the *trapz* function, we then calculated the area of the individualized D_2 peak (between 540 and 680 cm^{-1}), and that of the whole T-O-T signals (between ~230 and 680 cm^{-1}). This allowed the area of the D_2 signal to be calculated and compared to that of the whole T-O-T signal, as presented in Fig. 3D.

Molecular Dynamic Simulations

DLPOLY classic[39] was used to perform the MD simulation. The interatomic potential model was constructed by reference to that used to model silicate and alumina-silicate glasses containing rare earth or alkali ions[52,53]. Parameters for the Buckingham Potential $\phi_{12}(r) = A\exp(-Br) - C/_{r^6}$ are shown in Table S5.

Element 1	Element 2	A / eV	B / Å	C / eVÅ^6
O	O	2029.2204	0.343645	192.58
Al	O	12201.417	0.195628	31.997
Si	O	13702.905	0.193817	54.681
Na	O	4383.7555	0.243838	30.70
K	O	20526.972	0.233708	51.489

Table S5: Parameters for the Buckingham Potential.

In order to establish compensating ion clustering a 12,096 atom glass model was constructed for (Al/Si=1, X_K =0.75). To find the characteristics of alkali ions as a function of composition, five different compositions of alkali ions were studied ($X_K = \frac{K}{K+Na} = 0.00, 0.25, 0.5, 0.75, 1.00$) using smaller 1512 atom models, whose size was dictated by the computer-intensive diffusion and viscosity calculations. As the starting point, the crystal structure of natural nepheline $KNa_3Al_2Si_2O_{16}$ was taken from AMCSD (American Mineralogist chemical structure database) and Tait et al.[54] . The configurations of the other four compositions were derived by randomly replacing alkali ions in natural nepheline. Simulations were performed with 12,096 or 1512 atoms in at constant pressure/temperature (NPT) for all five ensembles. Temperature was controlled using Berendsen thermostats with a relaxation constant of 1 ps. Constant pressure was maintained by applying isotropic barostats with the same relaxation constant as thermostats. Initial heating runs of 500,000 production steps with 50,000 equilibration steps were performed using a time step of 1 fs in order to obtain a molten structure. 36 runs of 100,000 steps, with a decrease of temperature by 100 K between each were then performed to simulate the annealing process (cooling rate = 1 K/ps). The average Na-O and K-O distances were ~2.6 Å and ~3.0 Å respectively, replicating the bond lengths and CNs in natural nepheline. The corresponding respective ionic radii – 1.24 Å and 1.64 Å – were used in the molecular graphic representations in Fig. 4, showing the formation of clusters and channels as K^+ replaces Na^+. Another 10 melts

were simulated for compositions $(Na_{(1-XK)}\ K_{XK})_{0.167}\ Al0_{0.25}Si_{0.75}O_4$ (Al/Si=0.33) and $(Na_{\ (1-XK)}\ K_{XK})_{0.188}\ Al_{0.375}Si_{0.625}O_4$ (Al/Si=0.6), where X_K=0, 0.25, 0.5, 0.75, 1.

Analyses were performed with the glassy K_2O-Na_2O-Al_2O_3-SiO_2 structures for different mixed alkali compositions and different Al/Si ratios, each annealed to 300 K and with the melts at 2000 K. Additional calculations were made for mixed alkali melts with Al/Si rations of 0.33 and 0.67, as well as 1. Total and partial pair distribution functions for the glasses (Fig. 1D, 5A), were made as well as predictions of melt viscosity at 2000 K (Figs. 2, 5). The Vibrational Density of States (VDOS) was obtained from Fourier Transformation of the velocity autocorrelation function (Fig. 5C). Homonuclear and heteronuclear REDOR and Spin Echo magnetic second moment M_2 values (Fig. 6) were simulated from atomic distributions using the expression:

$$M_2 = A \sum_{j<k} \frac{1}{r_{jk}^6} rad^2/sec^2 , \qquad (S7)$$

where r_{jk} is the inter-cation distance averaged over the structure and A is a constant ~10^9 governed by the particular homo or heteronuclear combination[31].

Atomic viscosities were calculated from the atomic diffusivities D_i using the Eyring Equation $\eta_i = k_B T/D_i \lambda$, where λ is the atomic jump distance – the nearest distance between like neighbours. The atomic diffusivity D_i is given by $D_i = MSD/6t$, where *MSD* is the mean square displacement and *t* is the time. In order to access the diffusivities of network ions, MSD was calculated over 50 ns. Starting with diffusivities D_i for each element *i*, two different procedures were used: averaging over viscosities, $\eta = \sum_i c_i \eta_i$ and averaging over fluidities F_i, $\eta = 1/\sum_i c_i F_i$, where F_i=1/$F_i = 1/\eta_i$. The former gave good agreement with experimental values, while the latter grossly underestimated them.

Percolation theory analysis

For 3D geometries, the percolation threshold is defined by a critical value of the total volume of the objects $4\pi N r^3/3$, where *r* is their size and *N* their number, normalized to the size of the system L^3,

$$\xi_C = 4\pi N r^3/3L^3 . \qquad (S8)$$

For randomized octahedra[27] ξ_C = 0.252. The volume occupied by the charge compensating alkalis is defined by their ionic radii – Na^+ (1.24 Å) and K^+ (1.64 Å), and is normalized to size of the alkali system defined by the MD box size minus the volume of the network component, obtained from the ionic radii – Si^{4+} (0.25 Å), Al^{3+} (0.43 Å) and O^{2-} (1.36 Å). For $NaAlSiO_4$ ξ = 0.151 and for $KAlSiO_4$ ξ = 0.295, i.e. sodium clusters occupy a volume smaller than the percolation threshold while the volume occupied by potassium clusters lies above. Different alkali mixtures lead to the different overall total volumes, ξ, which are plotted in Fig. 5E, where ξ_C aligns with $N_K/(N_{Na}+N_K)$ = 0.71.

Like critical points, the percolation threshold is not a singularity, but the transition broadens as the size of the system reduces. In particular, if *p* is the probability of conduction within a cluster of chemical length *l* (i.e. the number of shells) and p_c and l_c the respective values at the percolation transition:

$$|p - p_c| = p_c/l \,. \qquad (S9)$$

Because the volume fraction ξ contains alkalis separated by approximately the same alkali-alkali distance $r_{M\text{-}M}$ ~3.4 Å (Figures 5A, S5), we assume $p \propto \xi$ and therefore that $|\xi - \xi_c| = \xi_c/l$, with the length of the cluster given by $l.r_{M\text{-}M}$. Taking the values of alkali volume fraction ξ from Figure 5E, values of the changing cluster length with X_K for different Al/Si compositions are shown in Figure S8. These compare with the MD viscosities of different Al/Si melts versus X_K (Fig. 5G) having similar super-exponential behaviour where $\eta \approx \log(l.r_{M-M})$. Notably, as alkali clusters grow in size, $l.r_{M\text{-}M}$, towards the percolation transition (Fig. 4), they increasingly immobilize the liquid network leading to similar super exponential growth in viscosity (Figs. 1B, 5G).

Violation of Lowenstein's Rule[25], established for most crystalline alumino-silicate structures for which there is an absence of Al-Al contacts, was quantified from $N_{Al\text{-}Al}/(N_{Si\text{-}Si}+N_{Si\text{-}Al}+N_{Al\text{-}Al})$ which equals approximately 3, 8 and 18 % for Al/Si=0.33, 0.6 and 1 glasses, respectively. $N_{T\text{-}T}$ values were determined from the areas under the respective inter-tetrahedral partial pair distribution functions $\rho(r)_{T\text{-}T}$.

Supplementary Discussion

The different structural roles of metallic cations in glasses

Binary $M^{x+}_{2/x}O\text{-}SiO_2$ glasses contain Si^{4+} *"network former"* cations, and $M^{x+}_{2/x}$ *metallic "network modifier"* cations, such as Na^+, K^+, Mg^{2+} or Ca^{2+}. The Si^{4+} is in tetrahedral coordination. The Si^{4+} tetrahedra bond with each other through their apical oxygens, which are in this case called bridging oxygens. Depending on the number *n* of bridging oxygens tetrahedra carry, different tetrahedral units, called Q^n units, are discriminated. The silica glass is mostly composed of Q^4 units, i.e. Si^{4+} is in tetrahedral coordination with 4 bridging oxygens, and hence, is strongly polymerized and its viscosity is the highest known. Modifier cations sever Si-O-Si bonds and form non-bridging oxygen atoms (e.g., see Dupree et al.[55]). The sodium silicate glass is composed of a mixture of Si^{4+} in tetrahedral coordination with 2 and 3 bridging oxygens, i.e. a mixture of Q^2 and Q^3 units. This profoundly depletes viscosity of the melt compared to that of pure silica.

In aluminosilicate glasses, metallic cations can also play the role of "*compensating*" cations. As aluminum Al^{3+} is present in the network as $[AlO_4]^-$ tetrahedral units, the negative charge deficit is provided by the compensating cations. Modifier and compensating cations concentrations depend on the $M^{x+}_{2/x}O/Al_2O_3$ ratio. When this is greater than 1, modifier cations will be predominant, and the proportion of compensating cations will be nearly equal to that of $[AlO_4]^-$ tetrahedral units. For $M^{x+}_{2/x}O/Al_2O_3 \leq 1$, as in this study, we can assume that all cations play a compensating role and the glasses are accordingly referred to as "*compensated glasses*". Modifier cations can exist in compensated glasses, but in very low proportions.

Na^+ and K^+ oxygen environments in aluminosilicate glasses

Na^+ ions in aluminosilicate glasses and minerals have a coordination number (CN) close to[23,54,56,57] ~7 ± 1, with an associated ionic radius of~1.21 Å[58]. From ^{23}Na spin echo NMR experiments (Fig. 6A), a small variation inferior to 3 ppm of the ^{23}Na MAS NMR barycentre with changing X_K from 0.00 to 0.73 confirms that Na-O distances barely change (only of 0.2-0.3 Å) with addition of K^+ [56,59], while Na^+ ions remain surrounded by a constant number

of Al^{3+} cations as shown by the absence of variations of the *ΔS/S₀* signal from {^{27}Al}^{23}Na REDOR NMR experiments (Fig. 6D). For a CN of 7, ionic radius of K^+ would be 1.54 Å, a radius even higher than that of O^{2-} anions in silicates (1.36 Å). In $KAlSi_3O_8$ or $KAlSiO_4$ minerals, K^+ CN is ~ 9 (48,51,54), with K-O distances of ~3 Å and a K+ ionic radius of 1.64 Å[58]. Na^+ CN is ~ 6 with Na-O distances of ~ 2.6 Å[54,60] in natural nepheline $K_{0.25}Na_{0.75}AlSiO_4$. Those values are replicated in the MD simulations (Fig. 5A) and agree with findings from X-ray absorption studies in K and Na alumino-silicate glasses[23,24]. They are used in the molecular graphics representations shown in Fig. 4, which reveal cluster and channel formation. High CN and M-O distances of K^+ (Fig. 5A) are associated with increases in T-O-T inter-tetrahedral distances (Fig. 3E, 5B, 5C). These may promote the enlargement of tetrahedral rings and cages, probably accompanied by a concentration of $[AlO_4]^-$ tetrahedral units around charge balancing K^+ cations, in conjunction with Loewensteinian disorder[25].

Mixed Alkali Effect (MAE) in silicate and alumino-silicate glasses and melts

Different alkalis mixed in oxide glasses retain their own particular environments[6,11] (Fig. 5A). This is the foundation of models of the MAE, explaining the minima[11,13] in ionic conductivity σ and diffusivity *D*. In oxide melts, the MAE is manifest by a minimum in viscosity[15] η, and in alumino-silicates melts by significantly asymmetric viscosity variations (Fig. 1B). Both phenomena can be understood in terms of micro-segregation of alkalis within the network with non-ideal mixing[13]. The σ minimum in the glassy state and the η minimum in the supercooled state of silicate compositions, each decrease as the temperature is raised but in different ranges – disappearing for the glass as the glass transition T_g is approached, and for the supercooled liquid as the melting point T_m is reached. Since nanostructured percolation channels frozen into the glass will first be established in the supercooled liquid as the temperature falls, they offer a common explanation of the MAE for σ, D and η respectively. While any mixed alkali minimum in η implies a maximum in *D*, assuming the Eyring equation relation $D = \frac{k_B T}{\eta \lambda}$, this so-called MAE anomaly in silicate compositions relates to alkalis migrating through channels within a frozen network below T_g, with a larger number of oxygens diffusing more advantageously through the network above T_g.

About the D_2 peak in Raman spectra of compensated glasses

Two contributions near ~570 and ~600 are observed in Raman spectra of compensated glasses (Fig. 3A and Figs. S1 and S2). They are respectively called the D_2^{SiAlSi} and D_2^{SiSiSi} signals in the following. Sharp signals in the 570-610 cm^{-1} portion of Raman spectra of compensated glasses can be assigned to breathing vibrations of three-membered rings (see Kubicki and Sykes[21] as well as discussion and references cited in Le Losq and Neuville[14]). The contributions of the D_2^{SiAlSi} and D_2^{SiSiSi} signals vary with the Al/Si ratio of glasses and with X_K (Figs. S1, S2): increasing Al/Si promotes the appearance of the D_2^{SiAlSi} signal, and increasing X_K promotes the D_2^{SiSiSi} signal. The D_2^{SiSiSi} signal is present in pure silica; it decreases with increasing Al/Si in K-bearing compensated glasses whereas the intensity of the D_2^{SiAlSi} signal increases (Fig. S2). Thus, D_2^{SiSiSi} can be assigned to breathing vibrations of three-membered rings only containing Si atoms, and the D_2^{SiAlSi} signal may be assigned to contributions of three-membered rings containing at least one AlO_4 tetrahedron. Molecular orbital calculations of vibrations in three-membered rings[21] show that three-membered rings with 2 or 3 Al atoms give vibrations at 544 and 526 cm^{-1}, while rings with only Si atoms give a peak at 610 cm^{-1}. Regarding this information as well as the constant frequency of

the peak we observe in the potassium alumino-silicate glasses and its clear separation from the D_2^{SiSiSi} peak (Fig. S2), the D_2^{SiAlSi} peak can be assigned to vibrations of three-membered rings containing one Al atom (at least).

In Fig. S2, the D_2^{SiSiSi} signal only forms a shoulder near 620 cm^{-1} on the Raman spectrum of the Al/Si = 1 product (see also Fig. 3A). This apparent increase of frequency is due to two things: first, the frequency of this shoulder does not represent the real position of the peak. Fitting spectra with Gaussian bands, we retrieved a frequency of 613 cm^{-1} for this peak, very close to its frequency in pure SiO_2 (606 cm^{-1}). Other frequency variations may arise from slight differences in the puckering of the three-membered rings. This is nearly non-existent in pure silica, where three-membered rings are planar. However, a slight puckering decreasing the T-O-T angle of the rings by only 1° will result in the observed shift of 7 cm^{-1} according to the model of Sen and Thorpe[20]. Such puckering may actually have a common origin with the formation of three-membered rings: those regions are formed following the creation of large tetrahedral cages and channels containing K^+ cation, and they may originate because of the introduction of stress due to volume effects.

Lowenstein's rule

The Lowenstein rule[25] stipulates that Al-O-Al bonds are avoided for energetic reasons in alumino-silicate minerals. Accordingly, alkali compensated glasses with Al/Si = 1 should present a network with regularly distributed Al and Si atoms, and therefore should contain only Si-O-Al bonds. Therefore, we would not expect to observe any signal from three-membered D_2^{SiAlSi} or D_2^{SiSiSi} rings in the nephelene (Al/Si = 1) glasses because such features imply the presence of Si-O-Si bonds (as such rings contain an odd number of tetrahedra while the bulk Al/Si ratio is 1), and thus of Al-O-Al bonds by compensation. However, this non-Lowensteinian feature is clearly present in mixed alkali glasses for Al/Si=1 (Fig. 3A; see also Fig. S1 for Al/Si = 0.72 glasses), pointing to Al-O-Al as well as Si-O-Si bonds within the network. MD simulations predict that, over the whole network, the significant presence of Al-O-Al correlations at around 18% for (Al/Si=1) (Fig. 5B), falling to 8% for (Al/Si=0.6) and 3% for (Al/Si=0.33) (Fig. 4).

Supplementary References

43. Neuville, D. R. & Richet, P. Viscosity and mixing in molten (Ca, Mg) pyroxenes and garnets. *Geochim. Cosmochim. Acta* **55,** 1011–1019 (1991).

44. Riebling, E. F. Structure of sodium aluminosilicate melts containing at least 50 mol% SiO_2 at 1500°C. *J. Chem. Phys.* **44,** 2857–2865 (1966).

45. Urbain, G., Bottinga, Y. & Richet, P. Viscosity of liquid silica, silicates and alumino-silicates. *Geochim. Cosmochim. Acta* **46,** 1061–1072 (1982).

46. Richet, P. Heat capacity of silicate glasses. *Chem. Geol.* **62,** 111–124 (1987).

47. Richet, P. & Bottinga, Y. Glass transitions and thermodynamic properties of amorphous SiO_2, $NaAlSi_nO_{2n+2}$ and $KAlSi_3O_8$. *Geochim. Cosmochim. Acta* **48,** 453–470 (1984).

48. Toplis, M. J., Dingwell, D. B., Hess, K.-U. & Lenci, T. Viscosity, fragility, and configurational entropy of melts along the join SiO_2-$NaAlSiO_4$. *Am. Mineral.* **82,** 979–990 (1997).

49. N'Dala, I., Cambier, F., Anseau, M. R. & Urbain, G. Viscosity of liquid feldspars. I: Viscosity measurements. *Trans. J. Br. Ceram. Soc.* **83,** 105–107 (1984).

50. Richet, P. & Bottinga, Y. Heat capacity of aluminum-free liquid silicates. *Geochim. Cosmochim. Acta* **49,** 471–486 (1985).

51. Richet, P. *et al.* Thermodynamics of open networks: ordering and entropy in $NaAlSiO_4$ glass, liquid and polymorphs. *Phys. Chem. Miner.* **17,** 385–394 (1990).

52. Okhotnikov, K., Stevensson, B. & Eden, M. New interatomic potential parameters for molecular dynamics simulations of rare-earth (RE= La, Y, Lu, Sc) aluminosilicate glass structures: exploration of RE3+ field-strength effects. *Phys. Chem. Chem. Phys.* **15,** 15041–15055 (2013).

53. Du, J. & Corrales, L. R. Compositional dependence of the first sharp diffraction peaks in alkali silicate glasses: A molecular dynamics study. *J. Non-Cryst. Solids* **52,** 3255–3269 (2006).

54. Tait, K. T., Sokolova, E. & Hawthorne, F. C. The crystal chemistry of nepheline. *Can. Mineral.* **41,** 61–70 (2003).

55. Dupree, R., Holland, D. & Williams, D. S. The structure of binary alkali silicate glasses. *J. Non-Cryst. Solids* **81,** 185–200 (1986).

56. George, A. M. & Stebbins, J. F. Dynamics of Na in sodium aluminosilicate glasses and liquids. *Phys. Chem. Miner.* **23,** 526–534 (1996).

57. Neuville, D. R., Cormier, L., Flank, A.-M., Prado, R. J. & Lagarde, P. Na K-edge XANES spectra of minerals and glasses. *Eur. J. Mineral.* **16,** 809–816 (2004).

58. Whittaker, E. J. W. & Muntus, R. Ionic radii for use in geochemistry. *Geochim. Cosmochim. Acta* **34,** 945–956 (1970).

59. Stebbins, J. F. Cation sites in mixed-alkali oxide glasses: correlations of NMR chemical shift data with site size and bond distance. *Solid State Ion.* **112,** 137–141 (1998).

60. Deer, W. A., Howie, R. A. & Zussman, J. *An introduction to the rock-forming minerals*. (Longman Group Limited, 1992).

Supplementary Figures

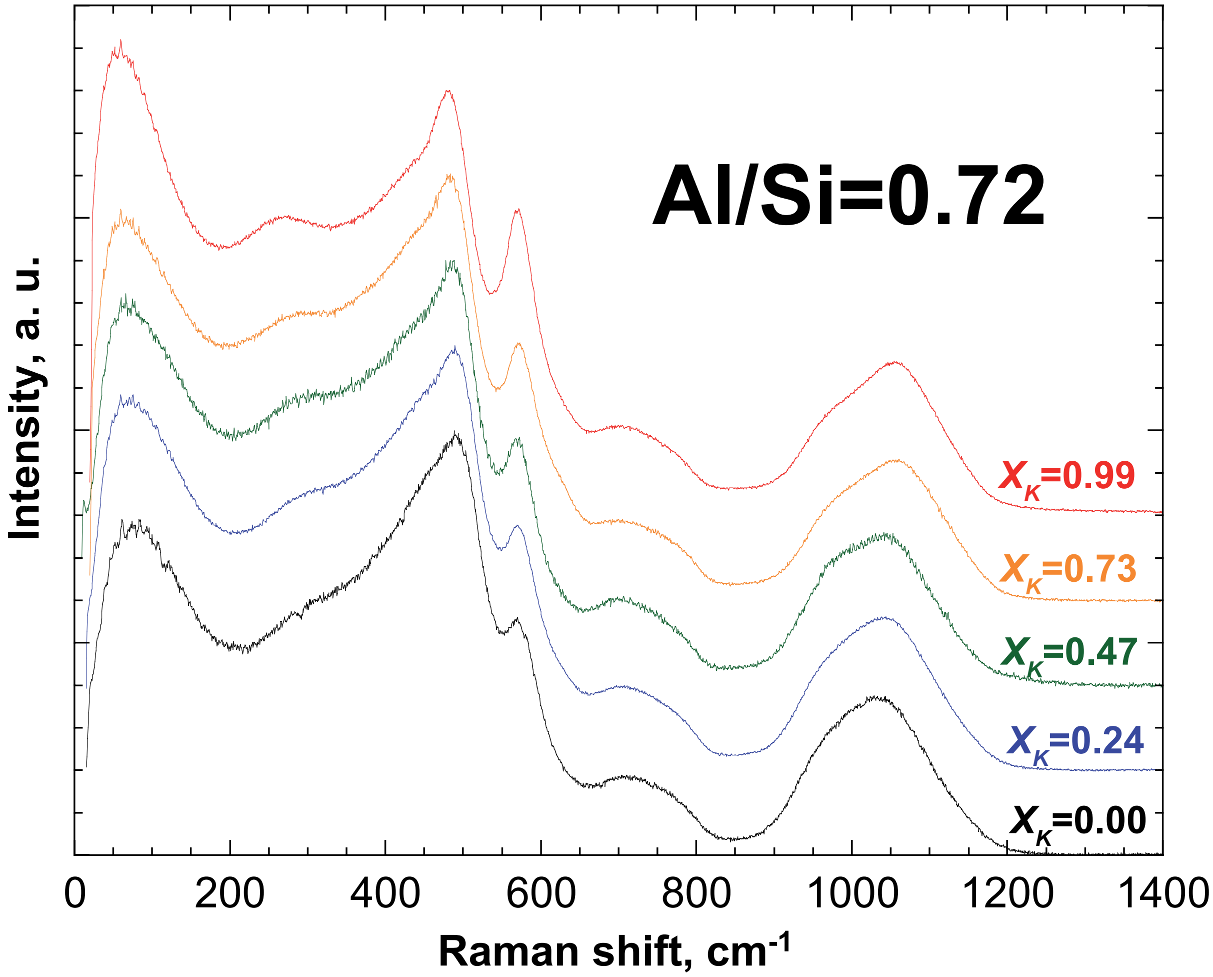

Figure S1: Full Raman spectra of the glasses with Al/Si = 0.72. An intensity increase of the D_2 570 cm^{-1} peak is observed with increasing X_K. Also, the frequency of the Q^n stretching band (~1000 cm^{-1}) and the intensity of the Boson peak (~70 cm^{-1}) both increase with X_K.

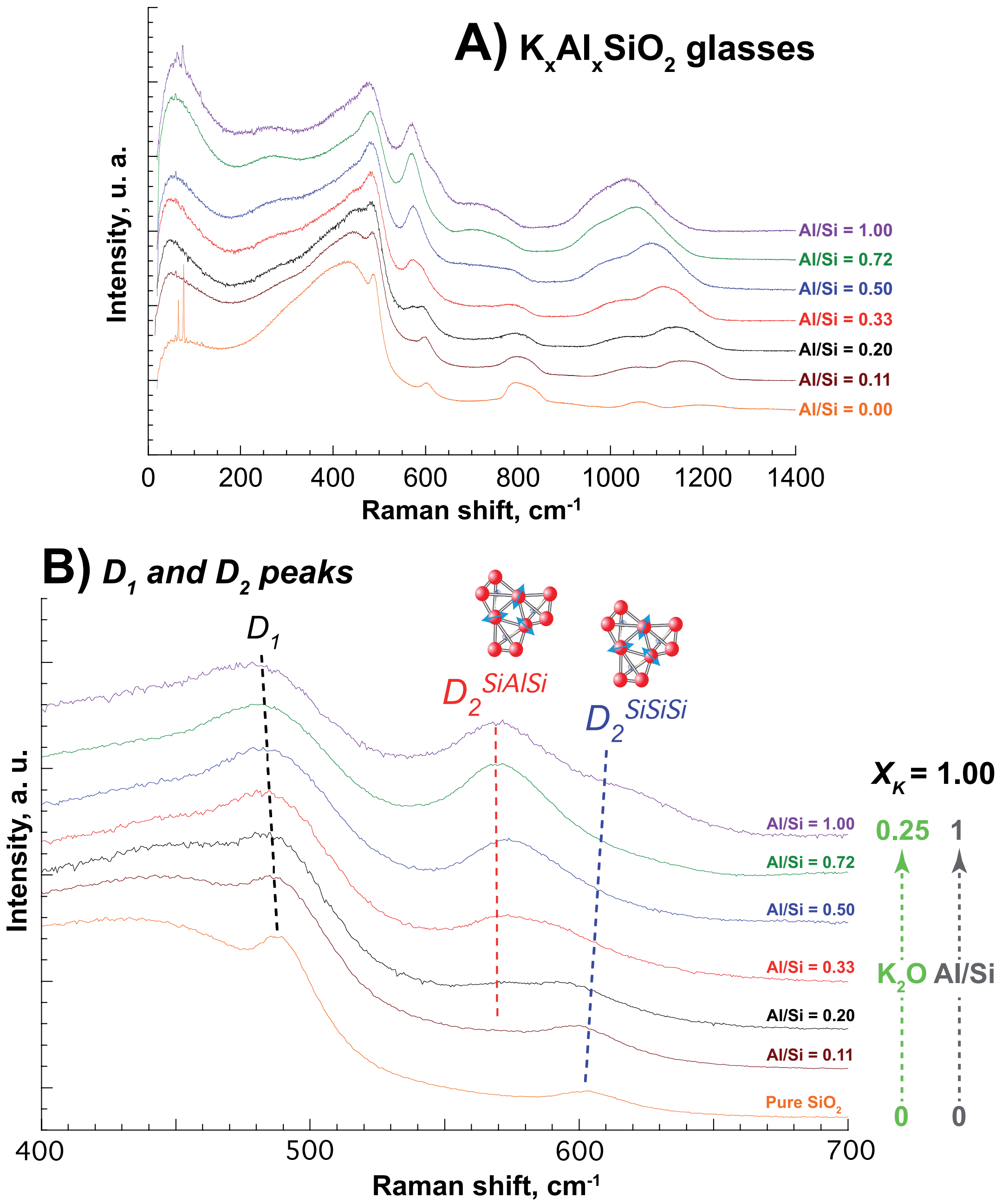

Figure S2: **(A)** Full Raman spectra of K^+ compensated glasses and of pure SiO_2. Presence of the Boson peak near 60 cm^{-1} indicates that all samples are glasses. A general decrease of the Raman shift of the high-frequency stretching band (between 1000-1200 cm^{-1}) is observed with decreasing SiO_2 concentration (hence increasing that of the Al_2O_3) and corresponds to a general decrease of the intertetrahedral angle. **(B)** Zoom on the 400-700 cm^{-1} Raman shift range. The peaks at 490, 570 and 600 cm^{-1} arise from breathing vibrations of four- (490) and three-membered (570 and 600) rings (see Supplementary Discussion). The D_2^{SiSiSi} signal is present in pure silica, whereas the D_2^{SiAlSi} appears with increasing the Al/Si ratio of glasses. Such variation indicates that the D_2^{SiSiSi} signal records breathing vibration of three-membered rings only composed of SiO_4 tetrahedra, whereas the D_2^{SiAlSi} records vibrations of three-membered rings that contain at least one AlO_2 tetrahedron. The small frequency increase of the D_2^{SiSiSi} contribution may result of ring puckering.

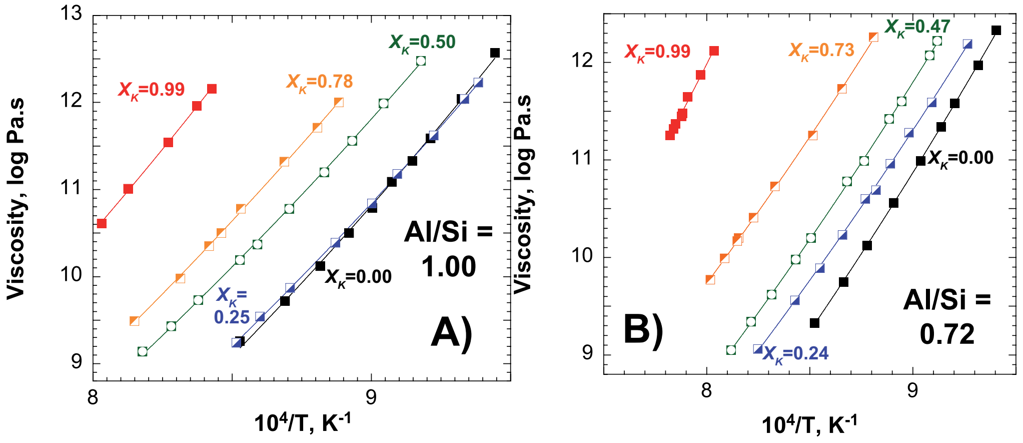

Figure S3: Viscosity (log Pa s) as a function of the inverse of temperature (K^{-1}) of the melts with Al/Si = 1 **(A)** and Al/Si = 0.72 **(B)**.

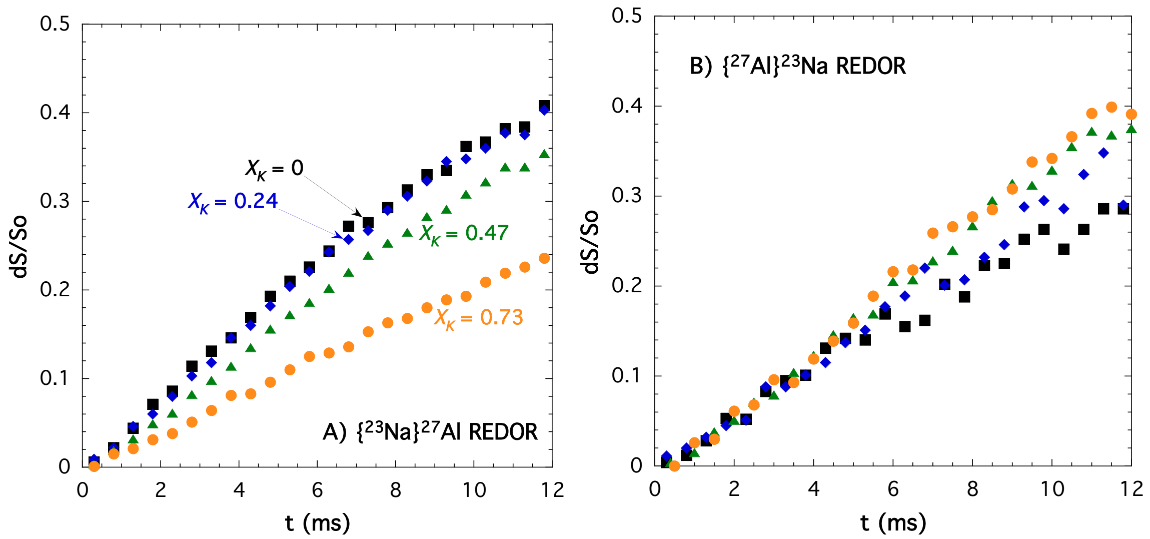

Figure S4: Normalized difference signal dS/S_0 versus dipolar evolution time *t* of the **(A)** {^{23}Na}^{27}Al and **(B)** {^{27}Al}^{23}Na REDOR MAS 20 kHz NMR data for glasses with Al/Si = 0.72. {B}A REDOR experiment probes the proximity of two nucleii A and B by looking at how the S spin echo of A is affected when the dipolar coupling constant between A and B is not cancelled by Magic Angle Spinning. *So* represents the "initial" spin echo of A with cancelling the dipolar coupling constant to B by MAS and S the observed attenuated spin echo when the A-B dipolar coupling constant is not cancelled by MAS, so that $dS = S - So$ measure the perturbation of the spin echo of A by the dipolar coupling to nucleus B. In our case, as we do not expect to have strong variations of the Al-Na distances with changing X_K, changes in the slope represents variations in the number of B nucleii in the environment of A, and the higher the slope, the higher B affects A.

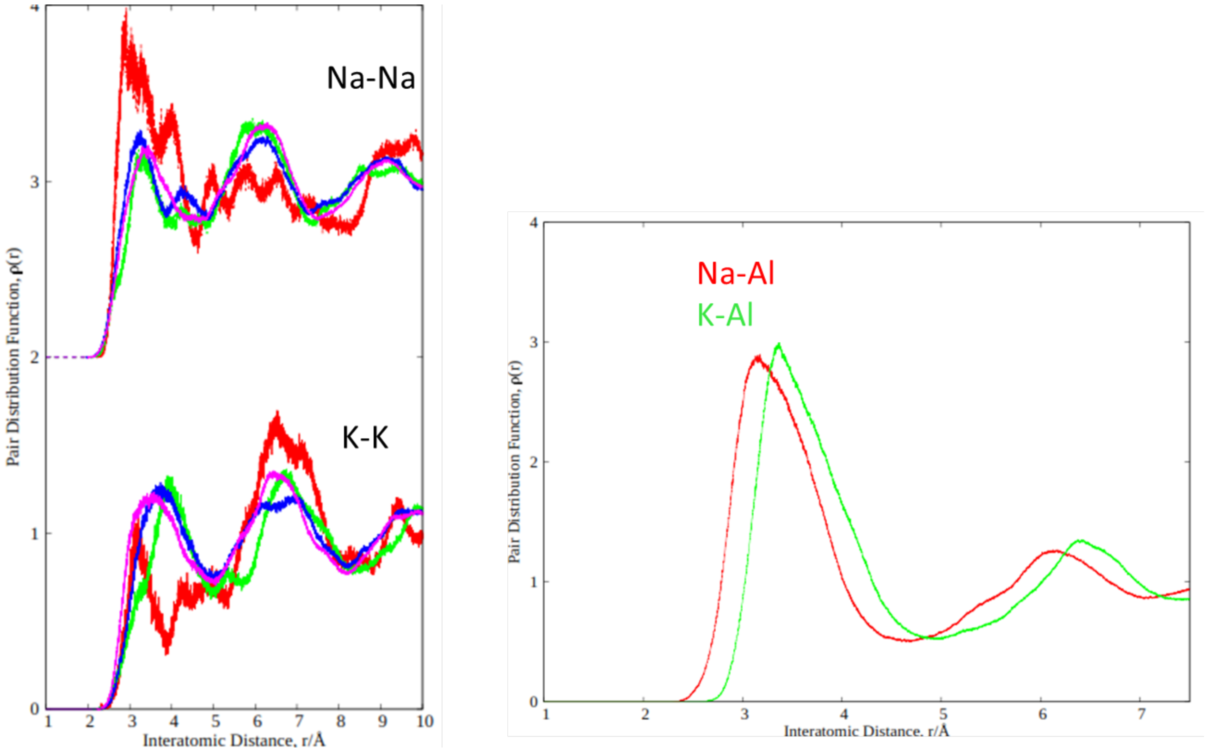

Figure S5: **(Left)** Na-Na (upper) and K-K (lower) partial pair distribution functions simulated for nephelene (Al/Si = 1) glasses containing different X_K = 0.25 (red), 0.5 (green), 0.75 (blue), 1 (magenta). These confirm the presence of alkali clusters visualized in Fig. 4D-I. **(Right)** Partial pair distribution functions for Na-Al in $NaAlSiO_4$ (X_K = 0, red) and K-Al in $KAlSiO_4$ (X_K = 1, green) glasses. Alkali-Al distances are clearly shorter for Na-Al than for K-Al, reflecting the higher ionic field strength of Na^+ compared to K^+.

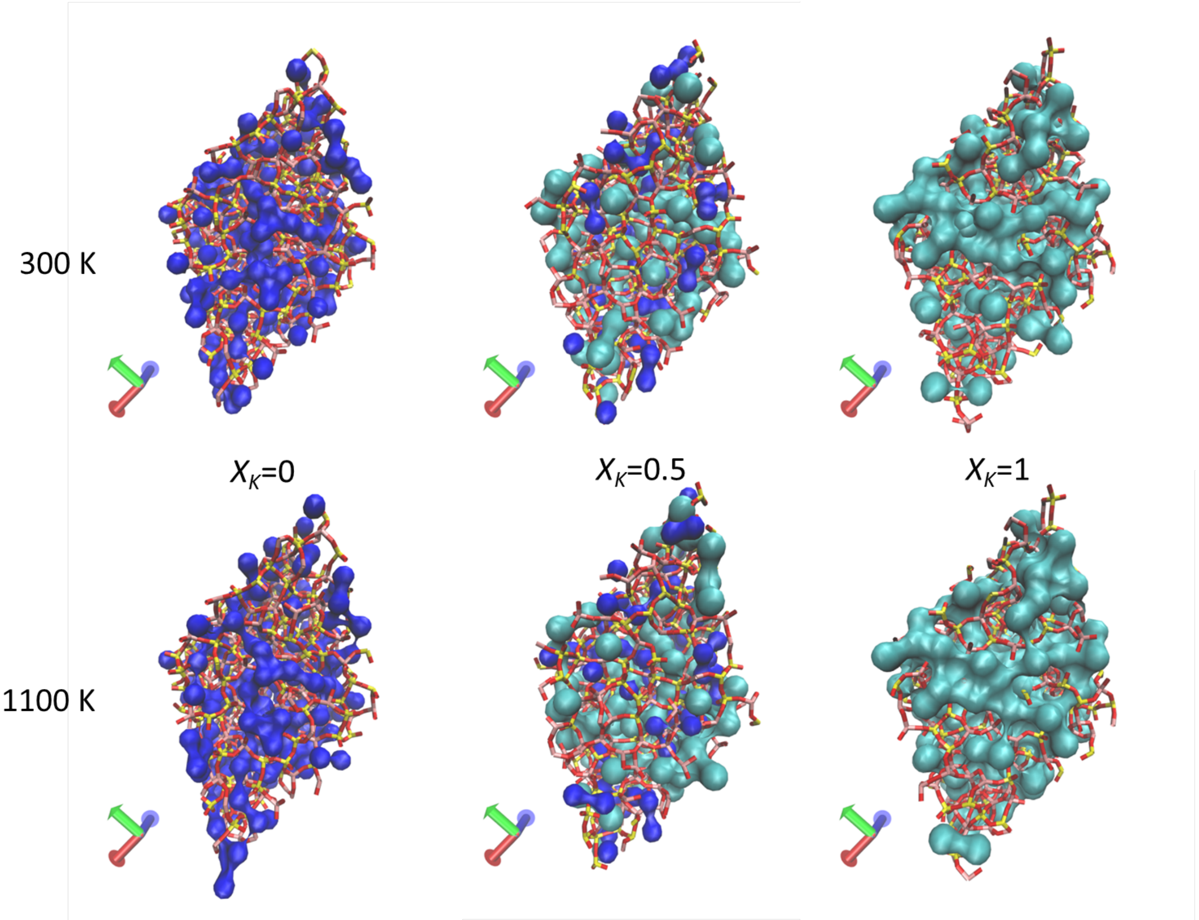

Figure S6: Micro-segregated alkali channels for $[Na_{(1-XK)}K_{XK}]AlSiO_4$ compositions at RT and at 1100 K close to the glass transition (Table S1), exhibiting not identical but very similar topology. The percolation threshold is achieved in the supercooled state and frozen in below T_g. At higher temperatures, this topology is lost.

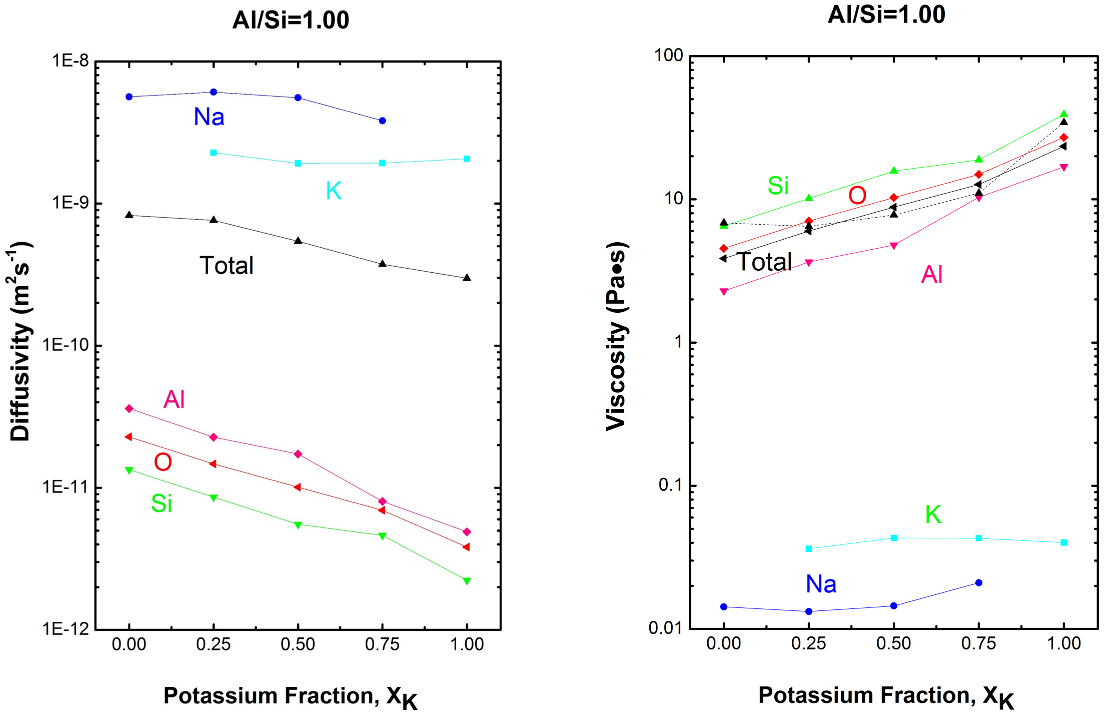

Figure S7: **(Left)** Diffusivity and **(Right)** viscosity for Al/Si=1 melts at 2000 K as a function of X_K. Diffusivities D_i of elemental contributions are shown, together with the corresponding viscosities η_i determined with the Eyring equation $\eta_i = k_B T/(D_i \lambda_i)$. The Total Diffusivity is $D = \sum_i c_i D_i$ and the Total Viscosity is $\eta = \sum_i c_i \eta_i$ which compares well with experimental values (Fig. 2A,B). Note MD values of η lie close to η_O.

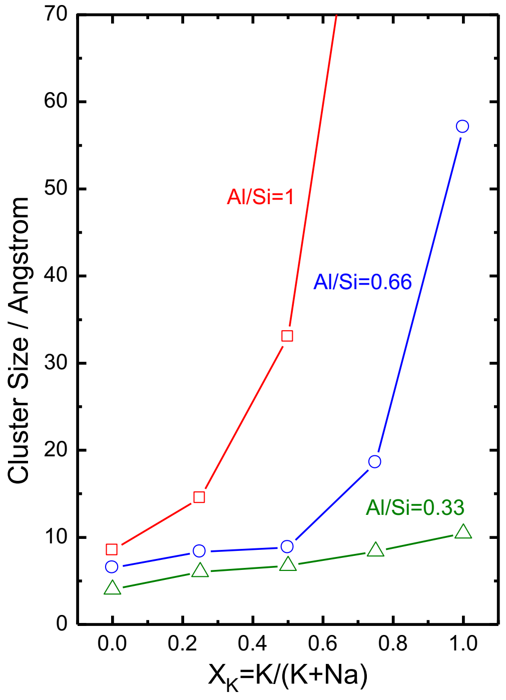

Figure S8: Cluster Size *versus* X_K for glass with Al/Si= 0.33 → 1, determined from alkali volume fractions given in Fig. 5E using $|\xi - \xi_c| = \xi_c/l$, where the cluster size is given by $l.r_{M\text{-}M}$, with $r_{M\text{-}M}$ the alkali-alkali distance ~3.4 Å. These values are contrasted with MD viscosity predictions in Fig. 5G, suggesting the approximate relationship $\eta \approx \log(\mathrm{l}.\, r_{M-M})$.
